# Biophysical larval dispersal models of observed bonefish (*Albula vulpes*) spawning events in Abaco, The Bahamas: An assessment of population connectivity and ocean dynamics

**DOI:** 10.1371/journal.pone.0276528

**Published:** 2022-10-20

**Authors:** Steven M. Lombardo, Laurent M. Chérubin, Aaron J. Adams, Jonathan M. Shenker, Paul S. Wills, Andy J. Danylchuk, Matthew J. Ajemian

**Affiliations:** 1 Harbor Branch Oceanographic Institute, Florida Atlantic University, Fort Pierce, FL, United States of America; 2 Bonefish and Tarpon Trust, Miami, FL, United States of America; 3 Florida Institute of Technology, Melbourne, FL, United States of America; 4 Department of Environmental Conservation, University of Massachusetts Amherst, Amherst, MA, United States of America; Japan Fisheries Research and Education Agency, JAPAN

## Abstract

Biophysical models are a powerful tool for assessing population connectivity of marine organisms that broadcast spawn. *Albula vulpes* is a species of bonefish that is an economically and culturally important sportfish found throughout the Caribbean and that exhibits genetic connectivity among geographically distant populations. We created ontogenetically relevant biophysical models for bonefish larval dispersal based upon multiple observed spawning events in Abaco, The Bahamas in 2013, 2018, and 2019. Biological parameterizations were informed through active acoustic telemetry, CTD casts, captive larval rearing, and field collections of related albulids and anguillids. Ocean conditions were derived from the Regional Navy Coastal Ocean Model American Seas dataset. Each spawning event was simulated 100 times using the program Ichthyop. Ten-thousand particles were released at observed and putative spawning locations and were allowed to disperse for the full 71-day pelagic larval duration for *A*. *vulpes*. Settlement densities in defined settlement zones were assessed along with interactions with oceanographic features. The prevailing Northern dispersal paradigm exhibited strong connectivity with Grand Bahama, the Berry Islands, Andros, and self-recruitment to lower and upper Abaco. Ephemeral gyres and flow direction within Northwest and Northeast Providence Channels were shown to have important roles in larval retention to the Bahamian Archipelago. Larval development environments for larvae settling upon different islands showed few differences and dispersal was closely associated with the thermocline. Settlement patterns informed the suggestion for expansion of conservation parks in Grand Bahama, Abaco, and Andros, and the creation of a parks in Eleuthera and the Berry Islands to protect fisheries. Further observation of spawning events and the creation of biophysical models will help to maximize protection for bonefish spawning locations and nursery habitat, and may help to predict year-class strength for bonefish stocks throughout the Greater Caribbean.

## Introduction

Connectivity of marine fish populations is driven by the complex interactions of biological processes and physical oceanographic conditions [1]. The exchange of biomass across spatially distant populations supports genetic diversity (genetic connectivity) and population growth and persistence (demographic connectivity) [2, 3]. As more connections within a meta-population are formed, local populations become more resilient to perturbations and yield increased abundances [4–6]. Delineating population connectivity and understanding the biophysical mechanisms that drive it can improve management and conservation strategies in species for which maintenance of metapopulation connections is critical. This process is becoming increasingly important as the global fisheries paradigm moves away from discrete stock-based management towards biologically defined stocks [7]. Furthermore, delineating connectivity can allow for structured decision making when establishing spatial management strategies, such as marine reserves [8, 9], challenging decisions in regions where resources are limited and enforcement is difficult [8, 10].

Many marine fish species form transient spawning aggregations, migrating from their home range to disparate locations to spawn [11, 12]. Identifying which local populations contribute to an aggregative spawning population is a significant component in the assessment of population connectivity [13, 14]. To do so, the full extent of the catchment area—the geographic extent of adults migrating from home ranges to spawning grounds and the extent of larval dispersal [15]—needs to be established. While local population connectivity through intermixing of spawning adults is important [13, 16], larval dispersal and the extent of dispersal has repeatedly been identified as the most significant driver of both genetic and demographic connectivity [1, 17, 18]. Dispersal is the holistic measure of connectivity, bridging spawning and recruitment events through the inclusion of transport—defined by the *x*, *y*, z displacement of larvae over time—and settlement [19]. The *in situ* assessment of larval dispersal source-sink dynamics in a marine environment is comparatively more difficult and labor intensive than tagging studies conducted on adult fishes [e.g., 17]. Field studies of larvae and their dispersal are often opportunistic, with region-specific goals and a broader focus on community composition [20–22]. Furthermore, follow up assessments of genetic relatedness [17] and/or comparing microchemical signatures with environmental concentrations [23] add time and complexity. As such, knowledge gaps often exist for information pertaining to both biological and physical controls of larval dispersal and connectivity.

Larval dispersal models (LDMs) are useful as a computational proxy for *in situ* monitoring of larval dispersal and movement, alleviating the logistical constraints of implementing field studies to fill knowledge gaps in larval dispersal/retention dynamics [24]. LDMs are coupled models with two modeling components, and an optional third: (1) Hydrodynamic model, (2) Lagrangian dispersal model, and (3) Individual based model (IBM). There are many regional hydrodynamic models maintained by working groups and governments, such as ROMS [25], SYMPHONIE [26], and NCOM [27]. These models numerically solve the Boussinesq hydrostatic equations of motion over a curvilinear grid to simulate the full water column ocean circulation and its hydrology. Lagrangian dispersal models build upon hydrodynamic models, implementing a particle tracking and settlement scheme for neutrally buoyant passive particles within the *x*, *y*, *z*, and time structure of the hydrodynamic model. Larval biology can be parameterized into the Lagrangian dispersal model, creating an IBM. IBM parameterizations include larval growth and changes in density, diel vertical migration, swimming ability, mortality, and restrictions on settlement habitat. In addition to the spatial tracking of particles over time, IBMs can also track environmental encounter histories and fate assignments [24]. Collectively, the three sub-models can be synthesized into a biophysical larval dispersal model that details the interactions between physical ocean conditions, biological processes, and settlement probability. Multiple biophysical larval dispersal modeling programs have been developed over the last two decades, such as LTRANS [28], CMS [29], Parcels [30, 31], and Ichthyop [32]. The increasing accessibility and useability of these models has brought together research communities dedicated to improving simulation accuracy and ease of implementation, broadening their application, and leading to model updates as new information is discovered.

Assessing connectivity has been central to establishing and supporting the management of bonefish. Some species of bonefish, such as *Albula vulpes* (herein bonefish), are an economically and culturally important sportfish that supports the catch-and-release flats fisheries throughout the Caribbean Sea and northwest Atlantic Ocean [33, 34]. The economic importance [35–37] and near threatened conservation status [38, 39] of bonefish have made the species of particular interest for conservation efforts. Extensive and numerous acoustic telemetry and conventional tagging studies informed by traditional ecological knowledge have revealed much of what is known about bonefish movement, and their findings are central to how the species is managed and protected. Adult bonefish home ranges are inclusive of intertidal sand and seagrass flats, mangroves, limestone hardbottom, saltwater creeks, and channels [40]. Recreational catch-and-release fishing is focused upon the flats habitats, and release handling practices are critical to post-release survival [41–43]. Additionally, significant progress has been made on bonefish spawning behavior. From October through April over 4–7 day periods spanning new and full moons, bonefish migrate from their shallow-water home flats to nearshore deeper-water locations and form pre-spawning aggregations (PSAs) of 2000–5000 fish [44, 45]. Currently, eight PSAs have been identified in The Bahamas [44, 46] and two in Belize [47]. Home ranges that contribute to the formation of a PSA can be up to 118 km away [14, 48]. From the nearshore PSA location, bonefish move offshore at dusk to spawn [44, 45, 49]. These tagging studies have helped to establish and support laws mandating catch-and-release fisheries [34, 50], community-based management [51, 52], and national park designations that protect vulnerable coastal habitats [48].

Limited information is available regarding the biology of bonefish larvae and the connectivity of bonefish populations through larval dispersal pathways. *A*. *vulpes* has an extended pelagic larval duration (PLD) and delayed settlement competency period, existing as a clear eel-like leptocephalus larvae for 41–71 days [53] before settling into littoral nursery habitats and metamorphosing into juveniles [54]. Zeng et al. [55] used an LDM for the first assessment of larval dispersal pathways for bonefish across the Caribbean, using eight known PSA locations and 18 theoretical locations. Larval dispersal restricted to the sea surface suggested significant connectivity of bonefish populations and supported the establishment of a Caribbean bonefish meta-population for management purposes. In the time since these simulations, significant discoveries have been made regarding bonefish spawning that can better inform bonefish LDMs. Lombardo et al. [49] documented bonefish diving to depths reaching 137.9 m after moving offshore to spawn at a depth associated with the pycnocline and thermocline. Furthermore, repeated offshore tracking of bonefish from the South Abaco PSA demonstrated that the selection of a pelagic spawning site is plastic [49]. Spawning depth, the association with frontal features, and variation in selected spawning location may have a significant effect on patterns of population connectivity and year-class strength.

Here, we apply a LDM to the observed spawning events documented in Lombardo et al. [49] in South Abaco, The Bahamas for the years 2013, 2018, and 2019. The incorporation of recent discoveries in bonefish spawning behavior and the biological parameterization of leptocephalus larvae provide a more refined representation of dispersal pathways and connectivity within the meta-population. As *in situ* pelagic and settlement stage larval sampling within the Bahamian Archipelago has not overcome the logistical constraints of regularized sampling efforts, larval development environments are undescribed for larvae successfully dispersed to islands throughout The Bahamas. The results of the LDMs represent a baseline estimation and description of dispersal pathways, connectivity within the region, and larval development environments.

Regional settlement densities and the persistence of connectivity are used to provide suggestions for spatial management strategies directed towards preservation of population connectivity within The Bahamas. *Post hoc* analyses of larval development environments during dispersal allowed for the evaluation of hypothesized contrasts for larvae dispersed to different islands. While larvae inherently experience different environments throughout the dispersal process, we hypothesized that (1) central measures of environmental conditions (mean temperature and salinity over dispersal duration) and (2) dispersal pathways (dispersal linearity, settlement days post spawn, release depth, and mean dispersal depth) of successfully settled larvae maintained relative relationships between islands across years (e.g., on average, larvae that settle on Abaco have a more circuitous dispersal path than those that settle on Andros). Interannual consistency in these relative relationships would be indicative of regional prevailing conditions that normalize larval dispersal, development, and connectivity.

## Methods

### Spawning events

The spawning events from which the suite of larval dispersal models were created from are published in Danylchuk et al. [44] and Lombardo et al. [49], which are the only detailed documented instances of bonefish spawning movements. Offshore spawning migrations from the PSA location in South Abaco were actively tracked with acoustic telemetry in November 2013, 2018, and 2019 [See 44, 49]. In 2013, five bonefish were gastrically tagged with Vemco continuous acoustic transmitters with pressure sensors rated to a depth of 50 m (V9P-2H, 9 mm diameter, 21 mm in length, 1.6 g in air, 2000 ms transmission period). In 2018 and 2019, six and four bonefish, respectfully, were surgically implanted with Vemco continuous pressure and temperature tags with a 250 m depth limit (V9TP, 9 mm diameter, 31 mm long, 4.9 h in air, period 1000 ms; Innovasea Systems Inc., Massachusetts). Not all tagged fish were successfully tracked offshore, as some fish emigrated, or were lost due to mortality or tag failure. The numbers of fish successfully tracked in 2013, 2018, and 2019 were one, three, and one, respectfully, and in 2019 the entire aggregation was also observed by sonar during the offshore migration. Active acoustic telemetry allowed for continuous monitoring of the migration into offshore waters that exceed 1000 m, and the use of pressure sensor tags logged depth information. The spawning events were described in four-dimensions: longitude, latitude, depth, and time. In 2019, CTD casts were taken over the three days preceding the spawning event and immediately upon observation of spawning behavior using a Castaway CTD rated to 100 m (SonTek YSI, Xylem Inc., New York).

In 2013 and 2019, spawning rush behaviors—a rapid ascent rate greater than the average moving rate—and mixing movements—balling and swirling behaviors when spawning [56]—were observed in the depth sensor data, indicative of the spawning events. However, gamete release over the full duration of the ascent or solely at the apex has not been confirmed. In 2018, the tag signals were lost during the initial descent in rough seas. Observations from the 2019 spawning event provided the most complete and detailed information about bonefish spawning ecology. The spawning event in 2019 revealed that bonefish spawning depth is likely associated with the pycnocline and thermocline boundary layer, as evidenced by the ejection of the acoustic telemetry tag [49]. Assuming an obligate relationship, thermocline depth was used to inform the parameterization of the 2013 and 2018 spawning events where the extent of the spawning rush below 50 m and the movement beyond the initial descent, respectively, were not observed (See Larval Dispersal Model below).

### Hydrodynamic model and grid

The hydrodynamic model chosen for this study is the Navy Coastal Ocean Model American Seas (NCOM AmSeas) [27] regional model from the Naval Oceanographic Office (NAVOCEANO). NCOM AmSeas is capable of resolving mesoscale and sub-mesoscale eddies, lending itself to applications in biophysical models [57–59]. The global NCOM model was developed by the Naval Research Laboratory and is based upon the Princeton Ocean Model with time invariant hybrid (sigma over z) vertical coordinates, allowing for higher resolution terrain following (sigma) and computational efficiency at deeper depths (z). NCOM AmSeas is a high-resolution derivative of global NCOM created in response to prediction and monitoring needs following the Deepwater Horizon incident [60]. The extent of the regional model covers the Gulf of Mexico and the Caribbean at 1/36° (~3.1 km) horizontal resolution and 40 depth levels. NCOM AmSeas ingests quality-controlled observations from the Navy Coupled Ocean Data Assimilation System (NCODA) [61], consisting of satellite sea surface temperature and altimetry as well as surface and profile temperature and salinity data. Boundary conditions are applied from operational global HYCOM (1/12° resolution) [62] and atmospheric forcings are provided by the Navy Global Environmental Model (NAVGEM) [63]. Tidal forcing is applied through two different methods. At the boundary, tides are imposed from the TPXO global inverse tidal prediction model [64]. Within the model domain, a tide-generating force is applied by incorporating astronomical forcing, ocean loading, and ocean self-attraction consistent with the TPXO model [see 65 for more detail]. NCOM AmSeas simulation archives consist of 3-hourly ocean state variables aggregated in NetCDF files including ocean temperature, salinity, sea surface height, east and northward flow velocity, and atmospheric forcing fields. Data were downloaded for each spawning season (October–April) and years (2013, 2018, 2019) observed with a spatial extent inclusive of potential dispersal to viable bonefish fisheries in The Bahamas, the northern extent of Cuba, and Florida and the Florida Keys.

All hydrography data transformations were done in MATLAB. To meet the hydrography data format and naming scheme requirements of Ichthyop, the NCOM AmSeas data were translated to a ROMS-like sigma coordinate grid. The bathymetry mask was created using the ROMS_TOOLS package [66] with bathymetry sourced from a subset of the ETOPO1 DEM [67], and clipped to only include depths greater than 1 m to eliminate land from the DEM. The shoreline mask was created from the Global Self-consistent, Hierarchical, High-resolution Geography database (GSHHG) [68]. Grid cells were masked when land comprised > 50% of the cell, single cell embayments, and single cell width edges along the domain boundaries. Spatial interpolation of the NCOM AmSeas data within the grid file was conducted using the ROMS_TOOLS package [66], which enforces mass conservation on the ROMS grid.

### Larval dispersal model

We selected Icthyop [32] as our biophysical LDM program due to its frequent co-application with NCOM AmSeas data in 3-dimensional particle dispersal modeling within the Caribbean [59, 69, 70]. A 3 h computational timestep was used, matching the NCOM AmSeas output time interval. The computational timestep did not exceed the fine-scale limit of the Courant-Friedrichs-Lewy criterion, approximated by $dt=0.7\times \frac{dGrid}{Umax}$, where *dt* is the timestep, *dGrid* is the average length of the grid cells, and *Umax* is the order of magnitude of the fastest current velocities in the hydrodynamic model [24, 32, 71]. A Forward Euler advection scheme was implemented with a horizontal dispersion rate (ɛ) set following equations (2 & 3) of Pelíz et al. [72]. Okubo [73] equation (4) was used to calculate the explicit Lagrangian horizontal diffusion (K_h_) for a 3.1 km grid. The horizontal dispersion is randomized at each timestep along a uniform distribution (δ ∈[−1,1]) [72], and the vertical dispersion is randomized using a vertical dispersion model [74] with cubic spline interpolation of the vertical diffusivity fields of the hydrodynamic model [32]. The randomized dispersion acts as a 3-D random-walk across multiple simulations, thus producing unique dispersal for each LDM iteration. To incorporate the stochastic variability of larval dispersal into the assessment of development environments, dispersal pathways, and connectivity, the LDM was run 100 times for each observed year, releasing 10000 larvae per iteration. The number of model iterations and larvae released were selected to minimize error in the potential or realized dispersal kernel, allow for representative sampling of the distributions governing the randomized directionality of the dispersion parameter enacted at each timestep, buffer against potentially significant mortality events (see Biology Parameterization), and to allow for within-year and across-year comparisons [24, 75, 76]. Furthermore, computational costs were considered when assessing the upper limit of how many larvae to release, as *post hoc* inspection would assess parameters governing every larva, at every time step, in each LDM iteration. Parameterizations for the simulation time, extent, stain (spawning event), transport, biology, and recruitment were set in the configuration XML file (see S1 File for 2019 model configuration XML).

### Spawning event parameterization

**Fig 1:**
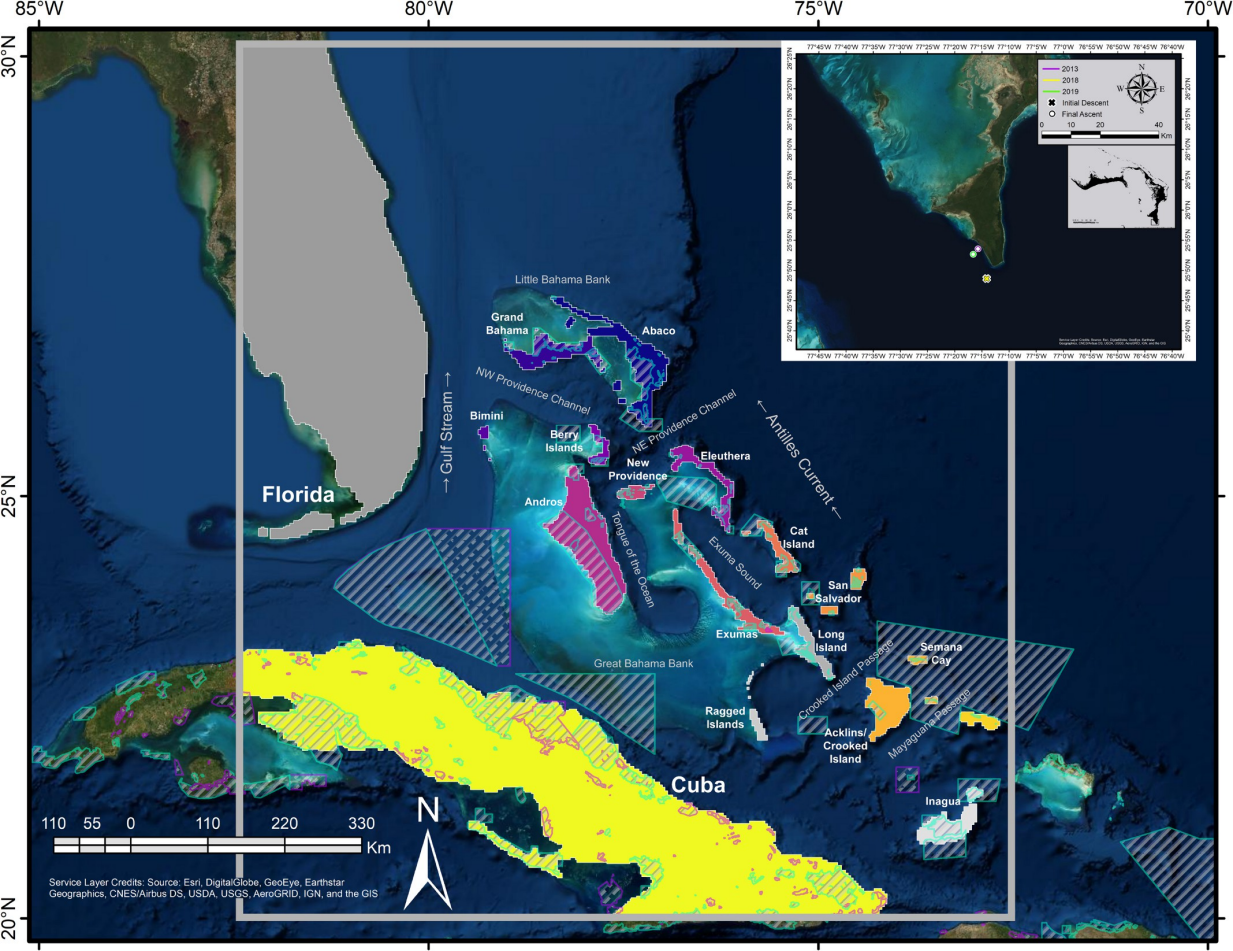
Settlement zone polygons and *A*. *vulpes* spawning locations. The domain of the hydrodynamic model was bounded within the gray box, inclusive of The Bahamas, Florida, and Cuba. Settlement zone polygons were classified per island group and cropped to the extent of the hydrodynamic model space. Island groups are color coded with settlement zones designated within the one to two grid cells (3.1–4.2 km) of the periphery, which has been extend from the shoreline of the land mask. Settlement zones shaded gray were not settled in any larval dispersal models. Inset are the putative spawning locations which correspond with the particle release locations. Prominent oceanographic features are labeled. Established protected areas are denoted with solid gray bars and teal outline, while proposed (2022) protected areas have broken gray bars and a violet outline. Protected areas were sourced from The Nature Conservancy Caribbean Protected Areas 05052022 (https://www.arcgis.com/home/item.html?id=6d15ba7e11a84278a13b40c8424db850t). The Esri “World Imagery” basemap layer and TNC Caribbean Protected Areas 05052022 layer are licensed under a Creative Commons Attribution-Noncommercial-Share Alike 3.0 License (http://creativecommons.org/licenses/by-nc-sa/3.0/).

The simulation start-time and location for each spawning event began at the time and location of the spawning observations in 2013 and 2019 or the last detection in 2018. Spawning events were observed on 17 November 2013 at 20:17 EST and 12 November 2019 at 06:00 EST, while on 20 November 2018 at 06:18 EST the final detections were received during the descent before being lost in rough seas. Spawning in 2013 and 2019 occurred west of the southern tip of Abaco, while in 2018 the fish were tracked south beyond the southern tip of Abaco (Fig 1). The simulation end-time of 71 d post-release coincided with the upper limit of the *A*. *vulpes* PLD and settlement competency period [53], allowing for the full spatial extent of potential dispersal. The range of spawning depths for each year were informed by observations in Lombardo et al. [49], where bonefish pelagic spawning habitat was coupled to the thermocline. *In situ* measurements of the water column were only taken by Lombardo et al. [49] in 2019. The *in situ* thermocline depth and the NCOM AmSeas thermocline depth were not equal, at 75.9 m and 50 m, respectively (See S1 Fig for NCOM AmSeas and CTD comparison). Therefore, the observed movement and spawning behaviors were translated into proportional depths in relation to the *in situ* thermocline. From these proportions, the spawning movements and behaviors could be related to the NCOM AmSeas thermocline depth so that the simulated spawning event depths would be at an equally proportional depth distance from the thermocline as the observed spawning event. In 2019, the upper observed spawning depth was 63.7 m, which was 84% of the thermocline depth (75.9 m). Therefore, the upper spawning depth for each simulated spawning event would be set at 84% of the NCOM AmSeas thermocline depth. The bottom extent of the spawning depth was determined by using the observed bottom extent of the 2019 spawn and the temperature data from the expelled acoustic tag, which functioned similar to an expendable CTD capable of returning data at depths greater than the depth rating of the Castaway CTD [See 49]. The bottom extent of the 2019 spawn coincided with a deviation from the consistent temperature-depth gradient, where the rate of cooling increased with depth (i.e., the maximum first-order derivative below the thermocline). Therefore, the bottom extent of spawning for each year was placed at the depth below the thermocline where the first-order derivative was maximized in the NCOM AmSeas data. These relationships resulted in simulated spawning depth ranges of 50.36–100 m, 50.36–125 m, 41.96–150 m for 2013, 2018, and 2019, respectively. Each spawning event released 10000 particles randomly distributed throughout the water column between the bottom and peak spawning depth with a horizontal radius of 150 m; equivalent to the 300 m detection range of the acoustic telemetry tags. Larval position, salinity, temperature, and vital status were recorded every 3 h.

### Biology parameterization

Little is known about bonefish leptocephalus biology and behavior throughout the pelagic duration of their development. While captively reared bonefish leptocephali displayed a vertical orientation and sporadic short burst swimming motions [77], these observations were only during the first < 10 days of the 71-day larval period, so volitional swimming behavior throughout development is not understood well enough to inform parameterization. Although burst and critical swimming velocities of other Elopomorph leptocephali have been experimentally measured and could be used in proxy, the ecology governing sustained swimming behavior throughout ontogeny is still not known [78]. Non-volitional movement may be imparted through specific gravity, or a particle’s relative density to seawater, which is likely to be important in leptocephalus dispersal as leptocephalus are uniquely buoyant [78, 79]. Larval density has not been measured for bonefish to parameterize buoyancy over development. However, density measurements over development have been taken for another Elopomorph, the Japanese eel *Anguilla japonica* [79], which we applied to bonefish by creating an equivalency between development time and the ratio of larval density to seawater density. The measured densities of individual *A*. *japonica* were converted to a proportion in relation to the density of water at the upper thermocline. Furthermore, the length of each individual *A*. *japonica* can be converted into a proportion of the time duration of larval development. Therefore, specific gravity measures for each pre-metamorphic larva by Tsukamoto et al. [79] were converted from a length:density ratio to $\frac{L/L_{\max}}{D_{l}/D_{s}}$, where *L* is the larva length, *L*_*max*_ is the maximum observed length of the pre-metamorphic larvae, *D*_*l*_ is the density of the larva at length *L*, and *D*_*s*_ is the seawater density at the thermocline. These proportions were matched to the larval duration of bonefish (See S2 File). Diel vertical migration (DVM) in bonefish is not well documented; however, most settlement competent larvae have been caught in the top 1 m of water when light traps were set in channels exceeding 4 m deep [53]. DVM was parameterized to maintain larval position within the top 2 m of water during the settlement competency period [41–71 days post spawn (DPS)]. The DVM programming is independent of spatial proximity to settlement zones, as there is no spatial component to the DVM parameters in Ichthyop. Lethal temperatures were parameterized with a lower bound at 14°C and upper bound at 32°C. Congener bonefish larvae have been captured in the Gulf of California at 15°C [80, 81] and in waters up to 30°C [21], while congener juveniles in the Indian River Lagoon, Florida have been observed in cold-snap mortality events at 14°C [82]. Transport behavior was parameterized to a *beaching particle*—the computational process of particle mortality due to contacting the shore before settlement competency. The biological basis for the beaching particle was not inclusive of larvae physically contacting the shore, as coastal hydrodynamic processes would deter such occurrences. Therefore, the beaching particle parameterization would be more appropriately described as *onshore mortality*. Onshore mortality encompasses mortality events driven by life stage-habitat mismatches (e.g., starvation, predation, inhospitable habitat) [83, 84], for which nearshore habitats inside of fringing reefs were presumed to be mismatched to pre-settlement competent bonefish larvae [53]. Additionally, the onshore mortality parameterization reduces the influence of unresolved nearshore processes over the full duration of dispersal that could not be captured at the grid resolution. Herein, the biological process of *onshore mortality* is synonymous with the computational process of the *beaching particle* parameterization.

### Settlement zones parameterization

Bonefish nursery habitats are associated with littoral habitats [54]. Settlement zones (Fig 1)—generalized settlement and nursery habitat equivalents that result in successful settlement if reached within the settlement competency period (41–71 DPS)—were created using ArcMap 10.6.1 (ESRI, Redlands, CA). Grid centroids were extracted from the hydrodynamic grid file in MATLAB and imported into ArcMap using the Make NetCDF Feature Layer tool. Grid points for each settlement zone were selected and converted into polygons using a 500 m buffer around the GSHHG shoreline polyline shapefile [68]. As grid points were centroids and not vertices, vertices were manually adjusted for the settlement zones to extend one to two grid cells beyond the shoreline of the land mask. Settlement zones were extended from shore to account for the potential ability of larval bonefish to maintain their position until biological and environmental conditions are suitable for settlement [85]; an ability that could not be parameterized into the biology of the larvae. Furthermore, the extension of the settlement zones ameliorates possible deficiencies in the hydrodynamic resolving of fine-scale shorelines processes. Shoreline composition was not discriminated against, as larval behavior and ability to navigate sub-optimal settlement habitats is unknown. Settlement zones from the shapefile created in ArcMap were converted to an XML file compatible with Ichthyop using MATLAB.

### Assessment of dispersal

#### Dispersal patterns and connectivity

All statistical analyses and visualizations were constructed in R 4.0.0 (R Core Team, Vienna, Austria). Outputs from the LDMs were aggregated by year then analyzed to describe annual and prevailing dispersal patterns. Larval dispersal patterns were visualized by calculating discrete gridded larval densities at each timestep, allowing for inspection and description of dispersal pathways and the final position of each larva at the end of the competency period at 71 DPS. Fate assignments—alive, out of domain, dead beached, dead cold, and dead hot—were logged at each timestep and the mean proportion of larvae within each fate classification was calculated from the aggregated LDMs. Proportional fate assignments throughout the dispersal period were used to provide context for gridded larval densities and insight for the temporal onset of abiotic sources of mortality. Settlement clustering was quantified using kernel density estimates (KDEs) from the MASS package [86], as larvae are independent, highlighting potential hotspots for recruitment. Island connectivity was quantified for each year using the percent of LDM iterations that resulted in successful settlement, the aggregated abundances of larvae settled, and the frequency of island combinations (hereafter settlement footprints) settled by LDM iterations.

#### Larval development environment

The larval development environment—inclusive of temperature, salinity, depth, and measure of dispersal deflection (hereafter coefficient of dispersal)—was summarized for each successfully settled larva and assessed for variability within years in relation to which island was settled. The coefficient of dispersal was calculated as the ratio of dispersal path and straight-line distances from the spawning location to the settlement location. The closer to 0, the more circuitous the dispersal route, and the closer to 1, the more linear the dispersal route. Larval development environment characteristics of successfully settled larvae were grouped by island and year and confirmed for non-normal distributions using a Kolmogorov-Smirnov test, which indicated non-normality for all measures. A Kruskall-Wallis test was then used to evaluate whether island was a statistically significant predictor of each of the larval development environment features. Pairwise comparisons were conducted using a *post hoc* Dunn’s test with Bonferroni correction. Pairwise comparisons were further evaluated for biologically relevant differences using Hedges’ g as a standardized measure of effect size. Hedges’ g is a sample size corrected measure of Cohen’s d and is more appropriate when comparisons involve samples sizes ≤ 50 [87, 88]. Hedges’ g is measured in relation to the pooled standard deviation of the two groups, and the strength of the effect was classified according to Cohen [89] as negligible (< 0.2), small (= 0.2), medium (= 0.5), or large (≥ 0.8). Confidence intervals were calculated using bootstrapping. Comparisons were conducted using the dunn.test package [90] and the effsize package [91].

## Results

### Dispersal patterns and connectivity

**Fig 2:**
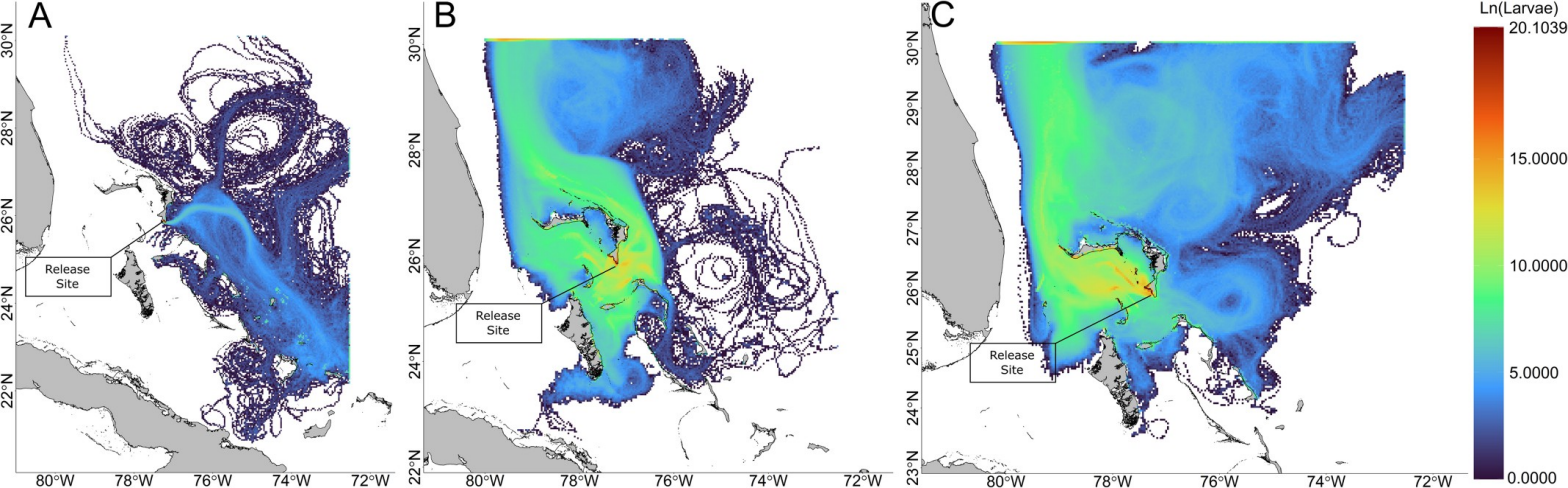
Larval density per grid cell at all time steps. Larval density per grid cell (natural log scaled) at all time steps. Data were aggregated across all LDM iterations. Panels correspond to (A) 2013, (B) 2018, and (C) 2019. Land-sea mask used for visualization is extracted from the NGA Global Shoreline Dataset. (https://shoreline.noaa.gov/data/datasheets/pgs.html). This work is licensed under a Creative Commons Attribution-Noncommercial-Share Alike 3.0 License (http://creativecommons.org/licenses/by-nc-sa/3.0/).

Discrete gridded larval densities of all 100 LDM iterations for each of the observed spawning years throughout the full extent of the PLD reveal the diversity in larval dispersal pathways across years (Fig 2). In 2013, the eastern current flow through the Northeast Providence Channel forced most pre-settlement competent larvae into the east-facing southern shore of South Abaco, resulting in onshore mortality and leaving a highly reduced number of larvae to be dispersed. Remaining larvae were transported eastward into the Atlantic where most larvae were transported south. Limited larvae successfully dispersed onto Great Bahama Bank at the southern extent by transport through Exuma Sound, the Crooked Island Passage, and Mayaguana Passage. Anomalous dispersal towards Cuba was observed via the Crooked Island and Mayaguana Passages. Gyre formations north and northeast of Abaco retained relatively few larvae without indication of settlement feasibility. Larvae that left the domain did so in the southeast direction.

In 2018, larvae exhibited more diverse dispersal patterns with strong pathway consistency exhibited by high gridded densities. Again, pre-settlement competent larvae experienced onshore mortality along the east-facing southern shore of South Abaco, though magnitudes less than in 2013, but also along the south-facing shore of South Abaco, resulting in onshore mortality. Larvae that did not experience onshore mortality proximal to the spawning location were mostly retained by eddies within Northeast Providence Channel and the large ephemeral gyre within Northwest Providence Channel. Strong northern transport from both Providence Channels was observed, with a substantial abundance of larvae exiting the model domain to the north-northwest, most of which originated from Northeast Providence Channel. Increased abundances on the north side of Grand Bahama indicate the presence of a larvae retaining eddy feature. Lesser southern transport was observed into the Tongue of the Ocean (TOTO), and fewer into the Exuma Sound. Eddy features retained larvae at both the northern and southern extents of the dispersal region, though it did not appear that larvae returned to the Bahamian Archipelago once circulated beyond its extent. Westward dispersal extended out to Bimini and was limited by the Gulf Stream, while eastward dispersal was controlled by Antilles Current dynamics. Few larvae were dispersed to the northern shore of Cuba via TOTO and traversing over the shallows of Great Bahama Bank.

The 2019 dispersal patterns were most similar to the patterns seen in 2018, with strong retention within the Providence Channels and large numbers of pre-settlement competent larvae experiencing onshore mortality along the south-facing shore in South Abaco. The majority dispersal pattern interacted with the large ephemeral gyre positioned within Northwest Providence Channel, with larvae transported westward out of Northwest Providence Channel and north along the Gulf Stream, exiting the model domain to the north. Larvae exited the domain over a vast area bounded to the west by the Gulf Stream and uninhibited to the east. Larvae did disperse south into TOTO and south along the Antilles Current, though their southern extent was limited to the eastern nearshore region of Andros and to the eastern nearshore region of Long Island. The western extent of dispersal reached Bimini and continued further west beyond its shore, and larvae uniquely occupied the area between Bimini and Andros. Similar to 2018, abundances north of Grand Bahama indicate an established eddy. In contrast to 2018, transport northward out of Northeast Providence Channel was not as significant as that from Northwest Providence Channel. Larval transport out of the domain was also more diffuse, and a gyre northeast of Eleuthera reduced the strength of transport south along the eastern shore of Eleuthera into Exuma Sound.

**Fig 3:**
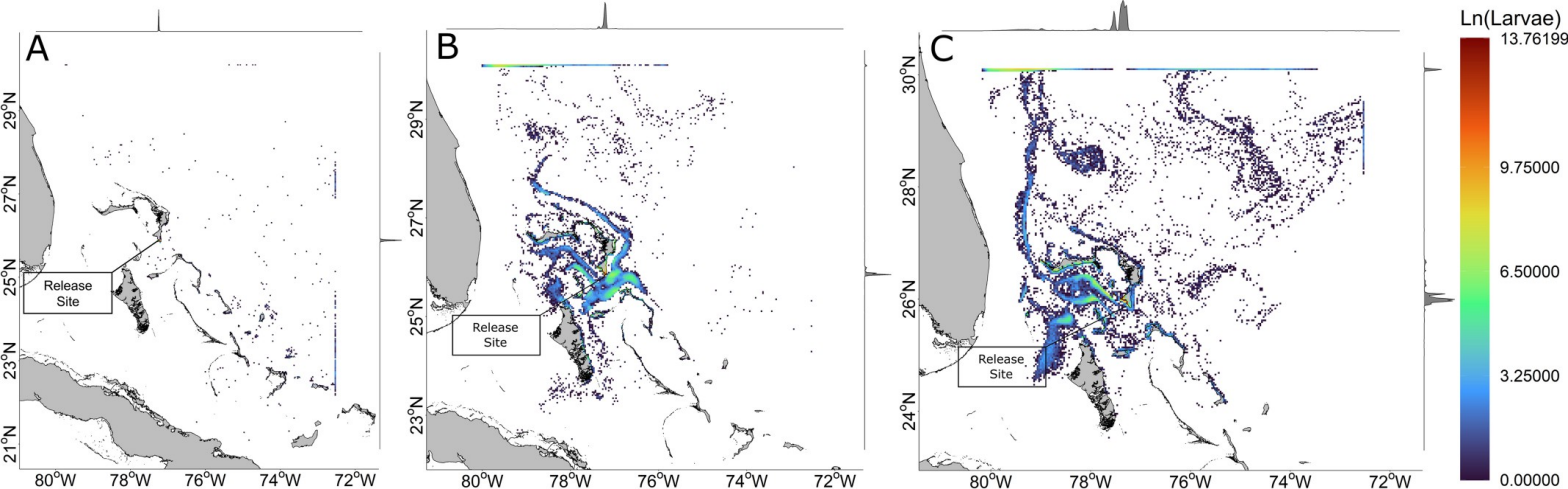
Larval density per grid cell at end of pelagic larval duration. Larval density per grid cell (natural log scaled) at final time step of the 71 d dispersal period. Data were aggregated across all LDM iterations. Density curves for larvae per grid are on the top and right axes. Panels correspond to (A) 2013, (B) 2018, and (C) 2019. Land-sea mask used for visualization is extracted from the NGA Global Shoreline Dataset. (https://shoreline.noaa.gov/data/datasheets/pgs.html). This work is licensed under a Creative Commons Attribution-Noncommercial-Share Alike 3.0 License (http://creativecommons.org/licenses/by-nc-sa/3.0/).

**Fig 4:**
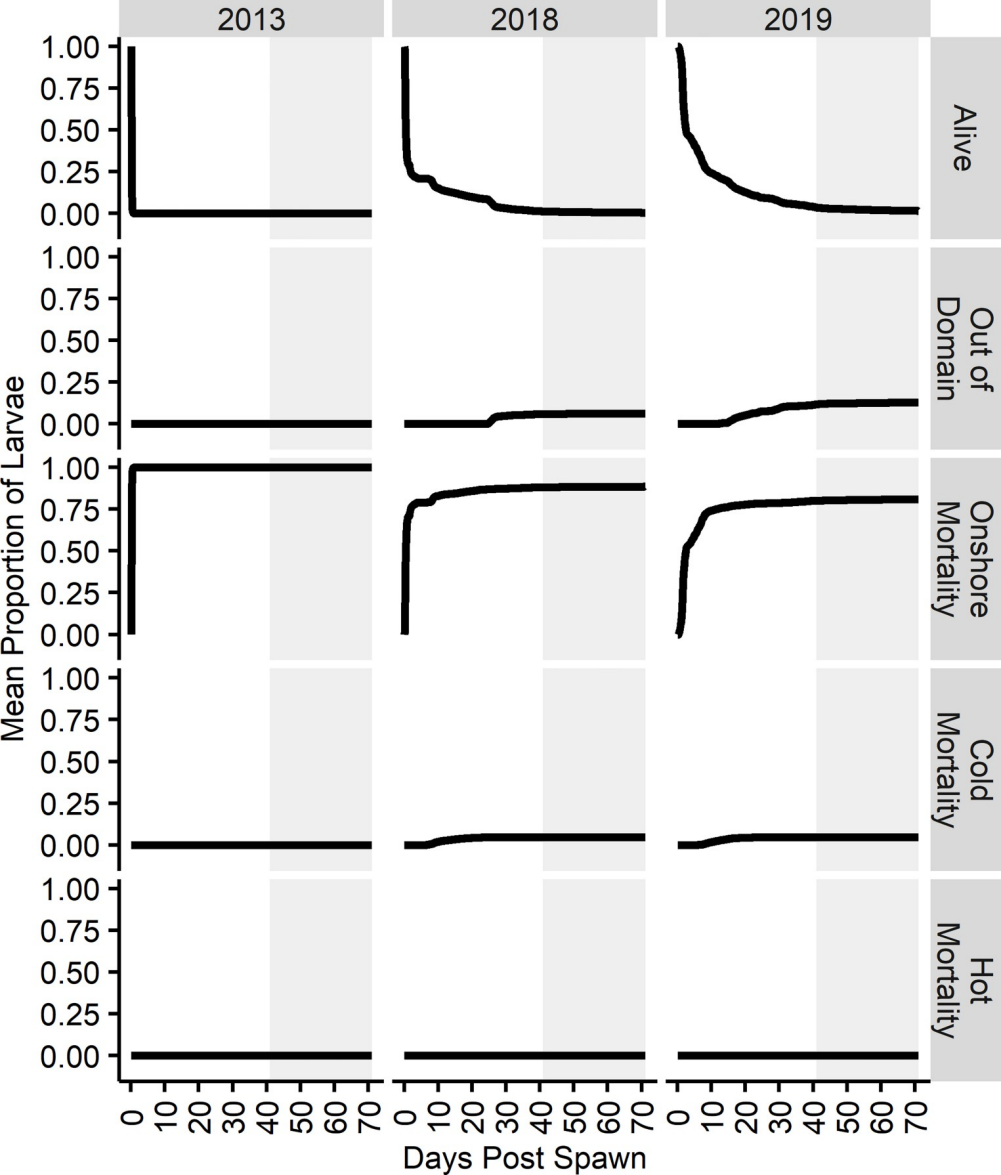
Mean proportion of survival and mortality fates throughout pelagic larval duration. Mean proportions per day of assigned fates—alive, out of domain, dead beached, dead cold, and dead hot—throughout the full 71 d dispersal period. Proportions on each day are an average of the proportion of larvae (n = 10000) assigned a given fate across all LDM iterations (N = 100). The settlement competency period is denoted by the gray box.

The final position of all larvae across aggregated LDM iterations in 2013 shows the highest density of larvae were found along the southern shore of Abaco, with 95% of larvae within one grid cell (Fig 3) as a product of pre-settlement competent onshore mortality (Fig 4). Larvae dispersed eastward beyond the model domain’s 72.5°W limit, with most leaving the domain southeastward. No apparent retention features were visible given the absence of clustering. In 2018, 46% of larvae were found within one grid cell along the southern shore of Abaco as a result of pre-settlement competent onshore mortality. Many larvae remained within the Northeast Providence Channel after 71 d, with larvae retained by the eddies within Northeast Providence Channel and the ephemeral gyre within Northwest Providence Channel. Larvae continued to be projected from the Northeast Providence Channel eddies in a northward trajectory, and a cluster of larvae were positioned within the shallow waters of Great Bahama Bank north of Andros. The majority of larvae left the model domain through the northern boundary. In 2019, 14% of larvae were found within one grid cell along the south-facing shore of South Abaco, mostly as a result of pre-settlement competent onshore mortality. Similar to 2018, larvae were retained within the Northwest Providence Channel ephemeral gyre, though in much greater numbers. Larvae continued to be projected from the gyre northward along the Gulf Stream. Larvae were also similarly clustered north of Andros, but over a much larger area spanning 100 km across. Large numbers of larvae were diffusely distributed throughout the sea northeast of the Bahamian Archipelago, and larvae exited the model domain over the full expanse to the north and northeast. The final positions of larvae across years were mostly determined by pre-settlement competent onshore mortality events (Fig 4). All years experienced ≥ 50% pre-settlement competent onshore mortality within the first day, with 95% pre-settlement competent onshore mortality in 2013. No heat mortalities were experienced in any year and cold mortalities were observed in 2018 and 2019, accounting for 4.7% and 4.6% of respective mortalities, with the majority occurring at the initial interaction with northeast edge of the Northwest Providence Channel ephemeral gyre. Larvae began to leave the domain earlier in 2019 than in 2013 or 2018, with the earliest departures always driven by the Gulf Stream.

**Fig 5:**
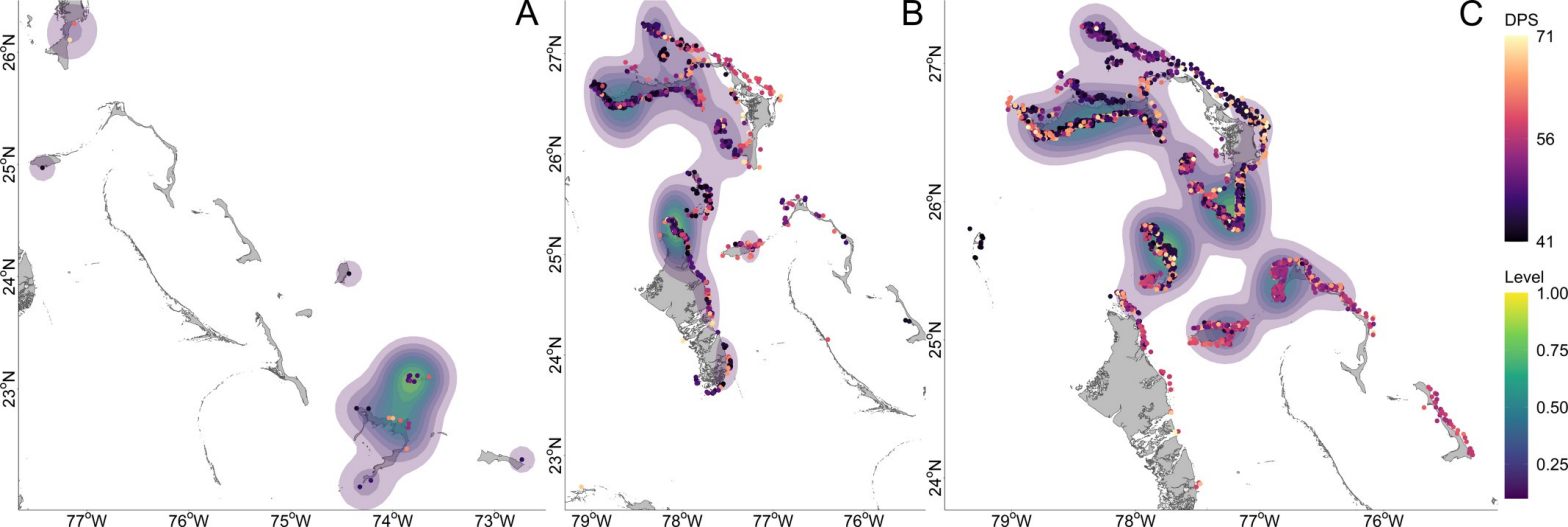
Kernel Density Estimates (KDE) for settled larvae. Kernel Density Estimates (KDE) for settled larvae for aggregated LDM iterations. Larvae are colored by settlement days post spawn (DPS), and KDE density estimates (Level) are standardized to a maximum value within a bandwidth of one. Core settlement areas colored yellow. Panels correspond to (a) 2013, (b) 2018, and (c) 2019. Land-sea mask used for visualization is extracted from the NGA Global Shoreline Dataset. (https://shoreline.noaa.gov/data/datasheets/pgs.html). This work is licensed under a Creative Commons Attribution-Noncommercial-Share Alike 3.0 License (http://creativecommons.org/licenses/by-nc-sa/3.0/).

Larval settlement was successful for < 0.01% of larvae in the aggregated 2013 LDM iterations, with five island groups settled upon. Settlement KDEs indicate that the core settlement distribution was located at the southern extent of The Bahamas, at Acklins/Semana Cay (Fig 5). In the aggregated 2018 LDM iterations, 0.12% of larvae successfully settled upon nine island groups with core settlement distributions located at the north end of Andros and west end of Grand Bahama. In the aggregated 2019 LDM iterations, 0.38% of larvae successfully settled upon eight island groups with core settlement distributions located at the southern end of Abaco, the Berry Islands, and mid-longitude of Grand Bahama.

**Fig 6:**
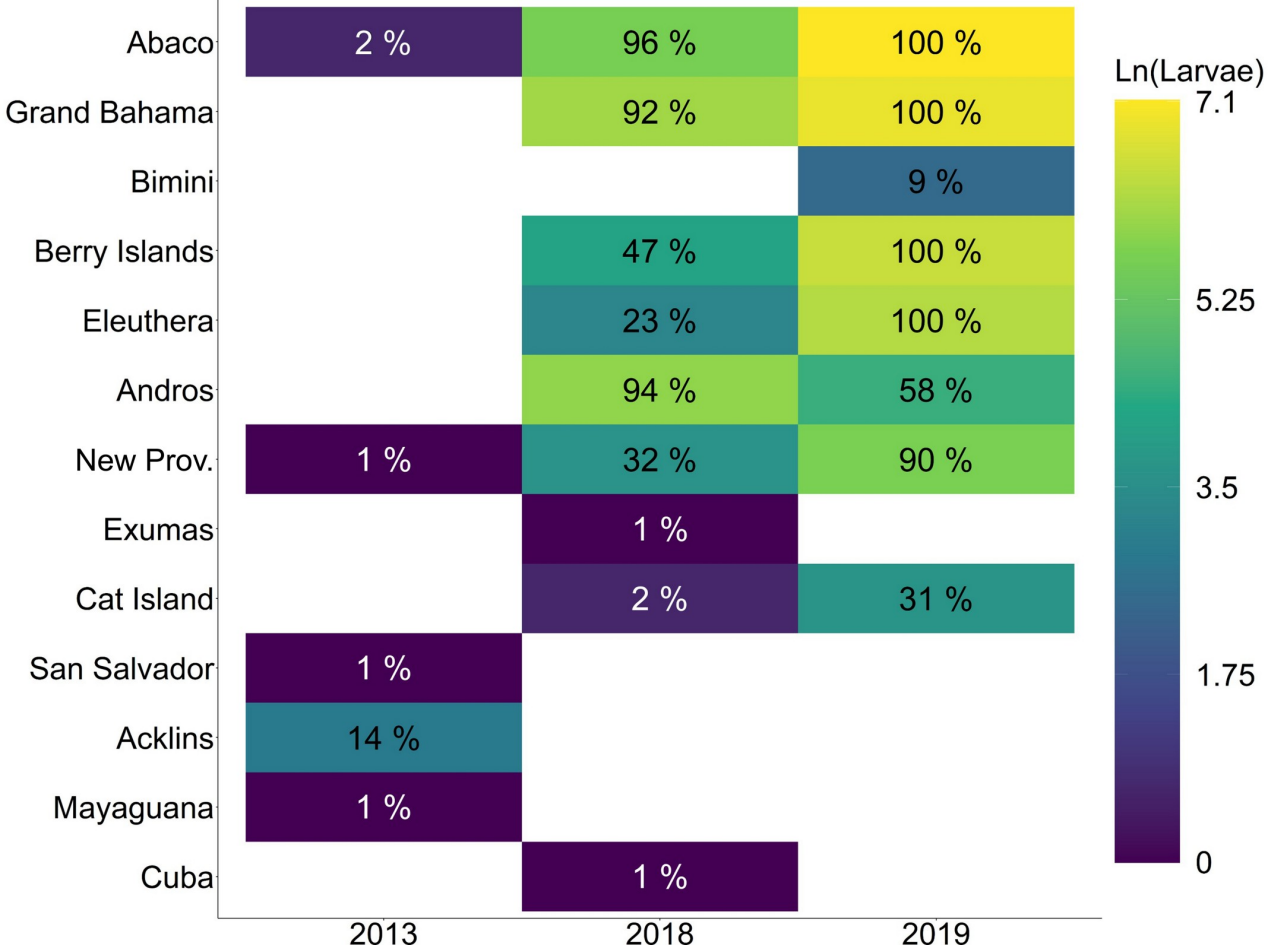
Larval connectivity matrix. Settled larvae (natural log scaled) per island over the full 71 d larval dispersal period. Count data were aggregated across all LDM iterations. The percent of LDM iterations that resulted in settled larvae are annotated.

Connectivity between Abaco and other islands varied across years, with the strongest connectivity showing island-level self-recruitment to Abaco and strong larval supply to Grand Bahama (Fig 6). The relationship between the number of larvae settled and the number of LDM iterations settled per island was positively monotonic. Connectivity in 2013 was especially poor given the high onshore mortality. The percent of 2013 LDM iterations which yielded successfully settled larvae was low, with larvae settling upon Acklins/Semana Cay most frequently at 14% of LDM iterations, though larval abundance was low. The most common 2013 LDM iteration settlement footprint—combinations of islands settled—occurred in 14% of the iterations and consisted of only Acklins/Semana Cay (S2A Fig). In 2013, no LDM iteration yielded more than 0.02% successfully settled larvae, which occurred in 4% of the LDM iterations, and the median percent of successfully settled larvae per LDM iteration was 0%.

Connectivity in 2018 was substantially stronger, with more consistent and abundant larval sourcing. The percent of LDM iterations settled in 2018 was nearly 100%, with Abaco settled most frequently at 96% of LDM iterations. Successful settlement in 2018 decreased with latitudinal distance from Abaco, as did abundances, though Eleuthera and New Providence had much lower settlement success than Andros. However, this could be attributed to differences in shoreline extents. Islands south of New Providence showed connectivity of little importance. The most common settlement footprint, at 22% occurrence, was Abaco, Andros, and the Berry Islands. In 2018, the median percent of successfully settled larvae was 0.1%, which occurred in 5% of LDM iterations, while the minimum of 0.03% occurred in 3% of iterations and the maximum of 0.23% occurred in 1% of iterations.

The 2019 spawn supplied the highest abundances and consistent sourcing of larvae. Four islands—Abaco, Grand Bahama, the Berry Islands, and Eleuthera—had larvae settle in 100% of LDM iterations, with both Andros and New Providence above 50%. The most common settlement footprint occurred in 32% of LDM iterations, included Abaco, Grand Bahama, the Berry Islands, Eleuthera, Andros, and New Providence. In 2019, the median abundance of successfully settled larvae was 0.37% and occurred in 3% of LDM iterations, while the minimum of 0.17% successfully settled larvae occurred in 1% of iterations, and the maximum of 0.58% successfully settled larvae occurred in 2% of iterations.

### Larval development environment

**Fig 7:**
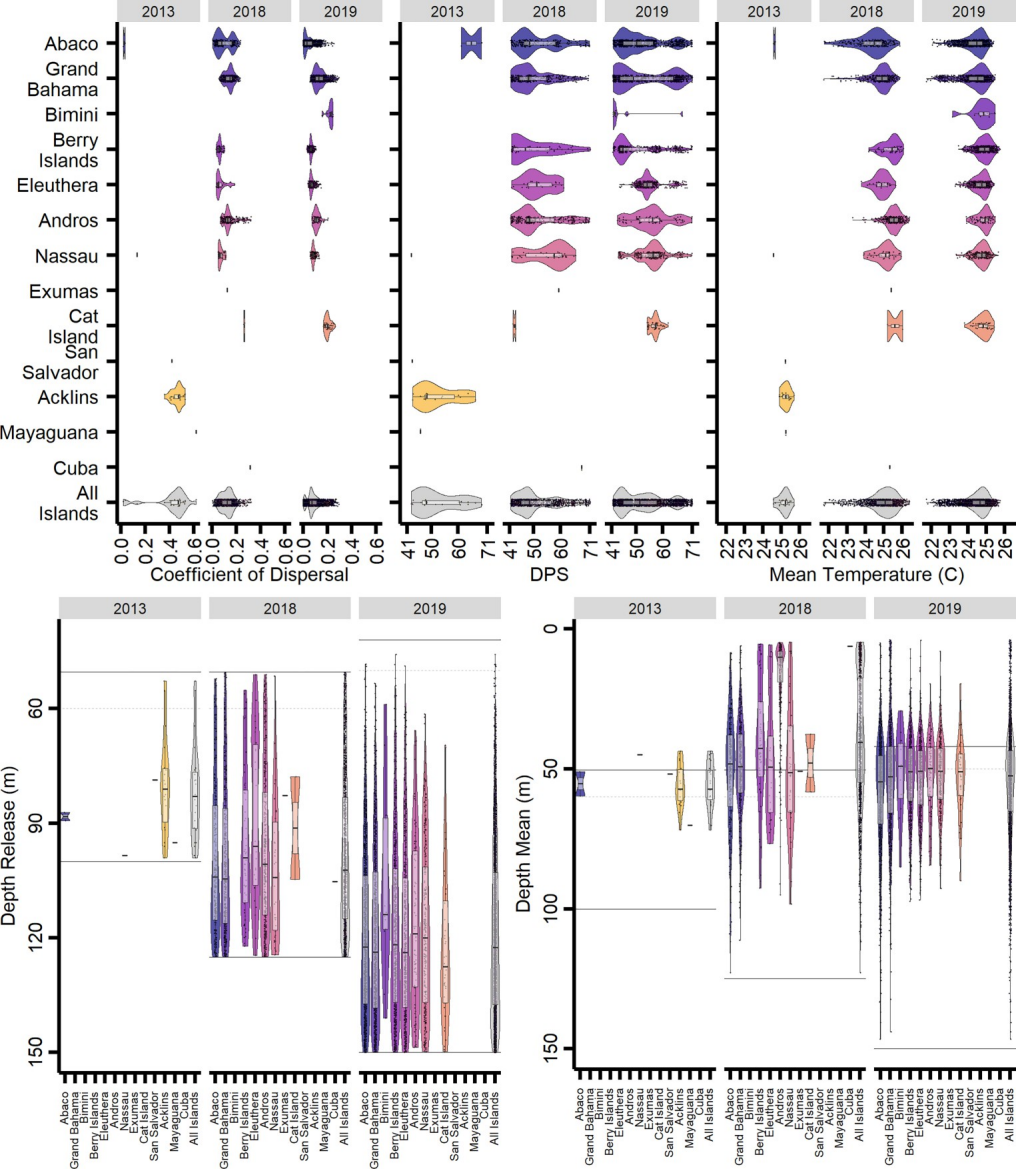
Larval development environment distributions. Larval development environment distributions by year, settled island group, and environmental parameter. Data were aggregated across all LDM iterations. Panels are detailed left to right, and top to bottom. (1) Coefficient of dispersal distributions for settled larvae per island. Coefficients of Dispersal tending towards zero indicate a more circuitous dispersal path. (2) Distributions of settlement time (days post spawn–DPS) for settled larvae per island. (3) Mean temperature history distributions over the duration of dispersal for settled larvae per island. (4) Release depth distributions over the duration of dispersal for settled larvae per island. Data were aggregated across all LDM iterations. Top and bottom spawning extents are indicated by black horizontal lines, and the NCOM AmSeas thermocline is indicated by the horizontal hashed gray line. (5) Mean dispersal depth distributions over the duration of dispersal for settled larvae per island. Data were aggregated across all LDM iterations. Top and bottom spawning extents are indicated by black horizontal lines, and the NCOM AmSeas thermocline is indicated by the horizontal hashed gray line.

**Fig 8:**
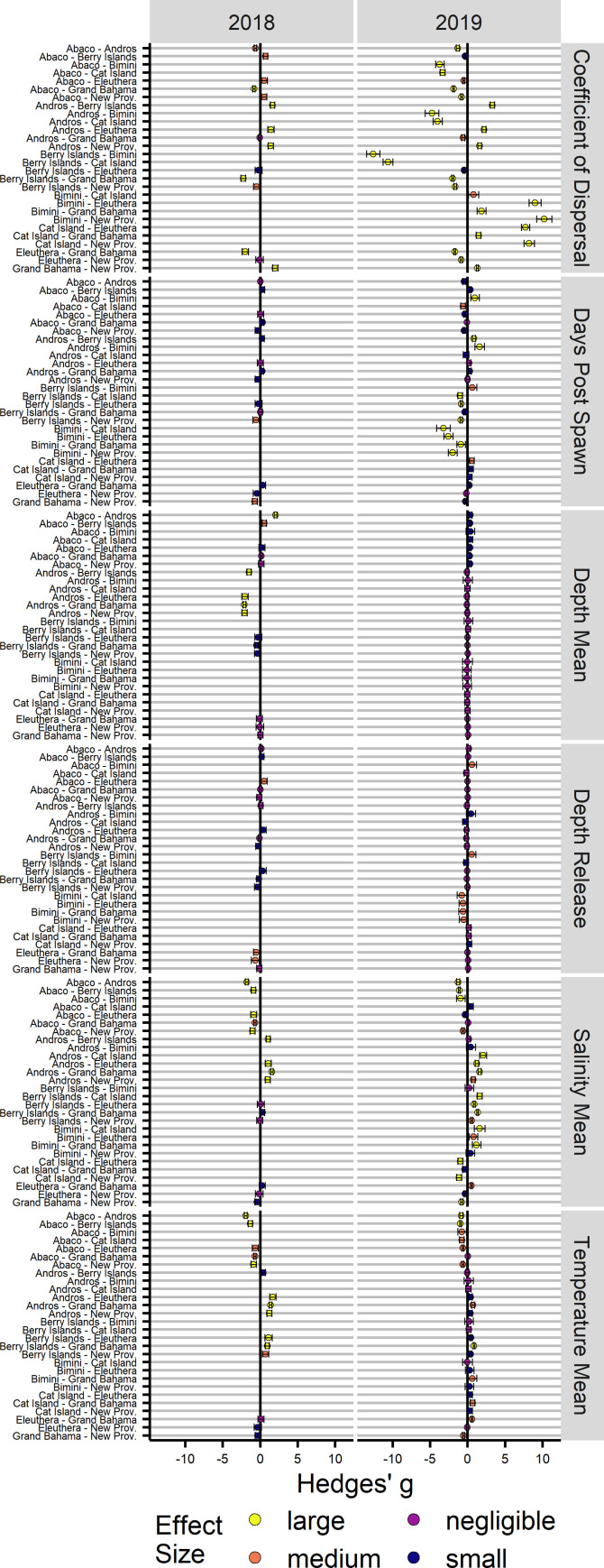
Larval development environment effect sizes. Effect size measures (Hedges’ g) for within year, paired settlement island comparisons of larval development environment characteristics. Effect sizes are classified according to Cohen [89] with exclusive bounds: Negligible (< 0.2), Small (< 0.5), Medium (< 0.8), Large (≥ 0.8). Comparisons are in relation to the first island listed, with negative values indicating less than. 95% confidence intervals are provided for each comparison.

Larvae were grouped by year and island settled to quantify the variability in development environments experienced and to assess whether relative relationships are consistent across years. Due to the nearly complete onshore mortality event of larvae from the 2013 spawn, statistical comparisons were only possible between the 2018 and 2019 spawns. Across years, larvae that settled more proximal to the spawning location had lower coefficients of dispersal, indicating retention in circulation features for longer distances (Fig 7). Successfully settled larvae ranged in their coefficient of dispersal from < 0.0001 to 0.620, equivalent to dispersal paths that were >1000 times to approximately 1.5 times the straight-line distance from the spawning location to the settlement location. The median coefficients of dispersal across years were 0.47, 0.13, and 0.07, respectively, with median dispersal path distances of 986.1 km, 1050.5 km, and 1057.5 km. Island specific coefficient of dispersal distributions were similar in shape and range in 2018 and 2019, which is indicative of similar or prevailing hydrodynamic conditions. However, there were differences in the coefficient of dispersal distributions for Abaco and Grand Bahama between years, as the gyre and eddy structures near the spawning locations were different in position, size, and numbers (S2E and S2F Fig). Larvae exhibited within year island level differences in coefficients of dispersal (S1A and S1B Table), with effect size differences in 2018 smaller than those in 2019 (Fig 8). Of the total 43 contrasts made within 2018 and within 2019, 36 had large differences with the difference in means ≥ 0.8 of the pooled standard deviation (sd) per comparison. Relative relationships between islands were largely conserved between the 2018 and 2019 spawns, with 73% of relationships maintained, indicating stable dispersal pathways.

Settlement most frequently occurred within the first third of the settlement competency period for all years, with settlement occurring throughout the 41–71 d settlement competency period (Fig 7). Settlement dates in 2013 ranged from 29 Dec 2013 to 24 Jan 2014 (42.5–68.75 DPS), nearly spanning the full settlement competency period despite low settlement success. The overall median settlement date occurred on 4 Jan 2014 (48.13 DPS) and was the earliest among the three years. In 2018, the settlement dates ranged from 23 Dec 2018 to 22 Jan 2019 (41.38–71.12 DPS), with the median date of 30 Dec 2018 (48.38). In 2019, the settlement dates ranged from 23 Dec 2019 to 22 Jan 2019 (41.38–71.0 DPS), with the latest median settlement date of 2 Jan 2020 (51.38 DPS). Median settlement dates among islands within years varied between 22, 25, and 15 d, respectively. Islands proximal to Abaco tended towards earlier peaks in settlement, and islands extending below the southern extent of the Berry Islands at 25.4°N reached the 50^th^ percentile of their total LDM aggregated abundance later than those above this latitude. Larvae exhibited within year island level differences in settlement DPS (S1C and S1D Table), with effect size differences in 2018 smaller than those in 2019 (Fig 8). Large differences in effect size were only seen within the 2019 spawn, with 10 comparisons having a difference in means ≥ 0.8 of the pooled sd per comparison. The large differences in 2019 were mostly comprised of comparisons with Bimini, where larvae dispersed to Bimini settled earlier than other islands. Relative relationships between islands were mostly conserved between the 2018 and 2019 spawns, with 53% of relationships maintained.

Larvae were released randomly throughout the parameterized spawning depths for each year and successfully settled larvae were produced from nearly the full range of depths. Across all years, larvae were more likely to successfully settle if released at a deeper depth (Fig 7). However, successful settlement did diminish at the bottommost extent of the spawning depths, which was parameterized to coincide with the depth below the thermocline where the temperature-depth gradient grew. Very few larvae released above the thermocline successfully settled in any year. Island was not significantly associated with release depths within either year (S1E and S1F Table), and no comparative effect sizes exceeded a Hedges’ g of 0.8 (Fig 8). The mean release depth of larvae dispersed to each island within years was statistically equivalent, with only seven of the 43 contrasts having confidence intervals that did not overlap with zero, most of which were comparisons with larvae that settled upon Bimini.

Larvae successfully dispersed over a wide range of depths across years, apart from the few larvae that settled from the 2013 spawn (Fig 7). Most successfully settled larvae were dispersed at a mean depth at or near the thermocline, though having been released below the thermocline and often much deeper. The distribution of mean dispersal depths in 2018 was broader than the distribution for the 2019 spawn, in part due to the heavily skewed shallow mean dispersal depths for larvae settling upon Andros in 2018. While mean dispersal depths were statistically different in within year, across islands comparisons (S1G and S1H Table), the mean dispersal depths only differed in effect size in 2018 when comparisons were made with Andros (Fig 8). As such, mean dispersal depths exhibited stable relative relationships, in that mean dispersal depths did not differ.

Mean salinities experienced by larvae over their dispersal did not vary more than approximately 0.6 PSU. Minimum and maximum salinities were also examined, though they collectively varied by only 2 PSU. Such minute differences given the euryhaline classification of Albulid larvae [92] did not warrant further investigation of differences in salinity exposure during the dispersal process or potential implications. Mean temperatures experienced by successfully settled larvae ranged from 21.73°C to 26.40°C (Fig 7), the coldest minimum temperature approached within 0.02°C of the 14°C cold mortality limit, and the warmest temperature was 28.95°C. Across all years and all larvae, the average range in temperatures experienced was 9°C, with a minimum and maximum deviation of 3.5°C and 14.4°C, respectively. The coldest mean temperatures were experienced by larvae dispersed to Abaco and Grand Bahama, with larvae experiencing warmer waters when dispersed to more southern locations. Mean temperatures were similarly ranged in 2018 and 2019, with the medians approximately at 25°C. Within year island comparisons were statistically different (S1I and S1J Table), and 15 of 43 contrasts having large differences in effect size (Fig 8). Differences were larger in 2018, though 67% of relative relationships maintained their directionality despite smaller differences among islands.

## Discussion

### Dispersal patterns and connectivity

#### Spawning site selection

The LDMs created in this study incorporated newly discovered ecological and biological information on the spawning movements and early life history of bonefish, building upon the first bonefish LDMs constructed by Zeng et al. [55] while taking a finer scale approach to understanding dispersal dynamics. The observed spawning events occurred within the same month across years, as bonefish exhibit spatial and temporal fidelity to PSA locations [45], with regular spawning occurring in Abaco each November around the full moon. Spatial fidelity does not extend to the spawning location [49] as hydrological conditions do and did differ. The active decision process on where to spawn in the three-dimensional pelagic environment has not yet been described. Inspection of flow vectors generated from the NCOM AmSeas data (S2D–S2F Fig) reveals the presence of gyre and eddy features within close proximity to the spawning locations in 2013 at 100 m and 2019 from the surface down to 125 m. It is reasonable to believe that bonefish are sensing circulation features, likely by current pressure differentials and temperature gradients. Unfortunately, tracking efforts in 2018 failed to maintain contact with the fish to their chosen spawning location; however, their anomalous extended offshore migration beyond the southern end of Abaco still supports the hypothesis of spawning site selection being reliant upon the presence of a circulation feature, as no circulation features were observed along their migration path and would warrant continued migration in search of such features. In the absence of circulation features, bonefish may select the spawning location based upon current direction and speed. Spawning location selection via these cues have been observed in other pelagic spawning fishes, such as bluefin tuna *Thunnus thynnus* [93], blue whiting *Micromesistius poutassou* [94], and many eel species that share an evolutionary history with bonefish [95]. Further efforts in bonefish active tracking and hydrographic sampling are needed to confirm these hypotheses.

#### Mortality

Flow direction, speed, and proximity to shore at the time of spawning significantly influenced the proportion of larvae available to settle. Pre-settlement competent onshore mortality within hours of the spawn was observed across all years, driven by spawning in close proximity to the western shore of South Abaco in the presence of an east flowing current. The significant and nearly instantaneous mortality may have been reduced given a finer grid system that could capture nearshore hydrodynamics; however, this LDM framework could not accommodate the nested grid systems that would be required, nor were such efforts feasible given computational costs and the size and complexity of the model domain. Furthermore, the 1/36° grid resolution already exceeded the resolution-domain relationships of most biophysical models within the literature [24]. The cessation of pre-settlement competent larval dispersal upon programmatic beaching/onshore mortality inherently reduces the influence of unresolved nearshore processes—inclusive of abiotic (hydrodynamics) and biotic (ecology)—by preventing continued or repeated exposure to nearshore regions of uncertainty. In doing so, significant losses of larvae may and did occur—the 2013 LDM resulted in 95% onshore mortality across all iterations. It is important to highlight the limitation of the LDM construct in that it is not feasible to parse the abiotic, biotic, and programmatic fractions of onshore mortality. Thusly, a tradeoff of uncertainty in dispersal for uncertainty in the source and true extent of onshore mortality is chosen when onshore mortality in enabled. Estimations in dispersal and environmental exposures will better align with the resolution and processes in which the hydrodynamic model was structured to capture. However, as this is applied to bonefish larvae, the absence of pre-settlement competent larvae in nearshore larval sampling where *Albula* spp. spawn supports that the pre-settlement competent larvae would not survive within region where nearshore hydrodynamic and ecological processes exert control on dispersal viability [20, 21, 53, 80], and thus the programmatic application of onshore mortality is suitably applied.

While these LDMs programmatically implicated physical contact with the shore as a cause of mortality to larvae, this is unlikely and without documentation. However, the mechanisms for mortality upon encountering a habitat before settlement competency are well documented. Habitat mismatch with the onset of endogenous feeding or thermal thresholds is a significant source of larval mortality [84], as is predation by planktivores in both pelagic and reef environments [83] that fringe the shores throughout The Bahamas. Furthermore, nearshore turbulence would likely lead to mortality in underdeveloped, fragile leptocephalus. Beyond thermal and domain boundary mortality, the multitude of sources for mortality in early-stage larvae were not possible to account for and parameterize within this LDM framework. Pre-settlement competent onshore mortality did appear to result in mortality at rates similar to other models that did incorporate mortality caused by mismatches in prey availability and predation [96], therefore the programing parameterization of beaching/onshore mortality may be useful as a proxy for mismatch driven mortality when parameterizations are unknown or not readily implemented into the LDM framework. Further work on the feeding ecology of bonefish larvae needs to be done to identify prey sources throughout the entire larval stage, as this information is unknown and would be valuable to incorporate into future LDMs as a spatiotemporal dispersal viability restriction.

The thermal tolerances of bonefish larvae are not well documented and can only be implied by capture data. We estimated the lower limit of thermal tolerance to be 14°C as informed by capture data of *Albula* sp. within the Gulf of California [80, 81] and mortality of juveniles within the Indian River Lagoon, FL, USA [82]—neither of which are *A*. *vulpes*. Cold mortality events within the 2018 and 2019 LDMs occurred early during dispersal at the initial interaction with the ephemeral gyre within Northwest Providence Channel. The cyclonic gyre subducted larvae from the deepest extent of the spawning range into deeper waters at approximately 250 m that reach 14°C. Bonefish larval dispersal and early life history is tightly coupled to interactions with gyre and eddy features as evidenced by their extended pelagic larval duration. The observation of gyre mediated thermal mortality for larvae spawned at the deepest extent of spawning rushes introduces a mechanism for limiting the depth at which spawning rushes begins for bonefish. Behavior implicated in sensing the boundary of preferred spawning habitat was observed through acoustic telemetry data, where bonefish oscillated across depths, seemingly to inspect hydrologic conditions throughout the offshore migration process [49]. Such behaviors allow bonefish to target the thermocline at the upper limit of the spawning rush and also appears to appropriately limit the bottom extent of the spawning rush to reduce mortality, as evidenced by the reduction in larval settlement success at the bottom most extent of the spawning rush (Fig 7; S2G Fig). Conversely, no upper thermal mortalities occurred in any year. However, the upper thermal tolerance for bonefish larvae should be investigated given predicted climate scenarios that will increase heat retention in surface waters and potentially destabilize current patterns and winter stratification [97].

#### Dispersal pathways

**Fig 9:**
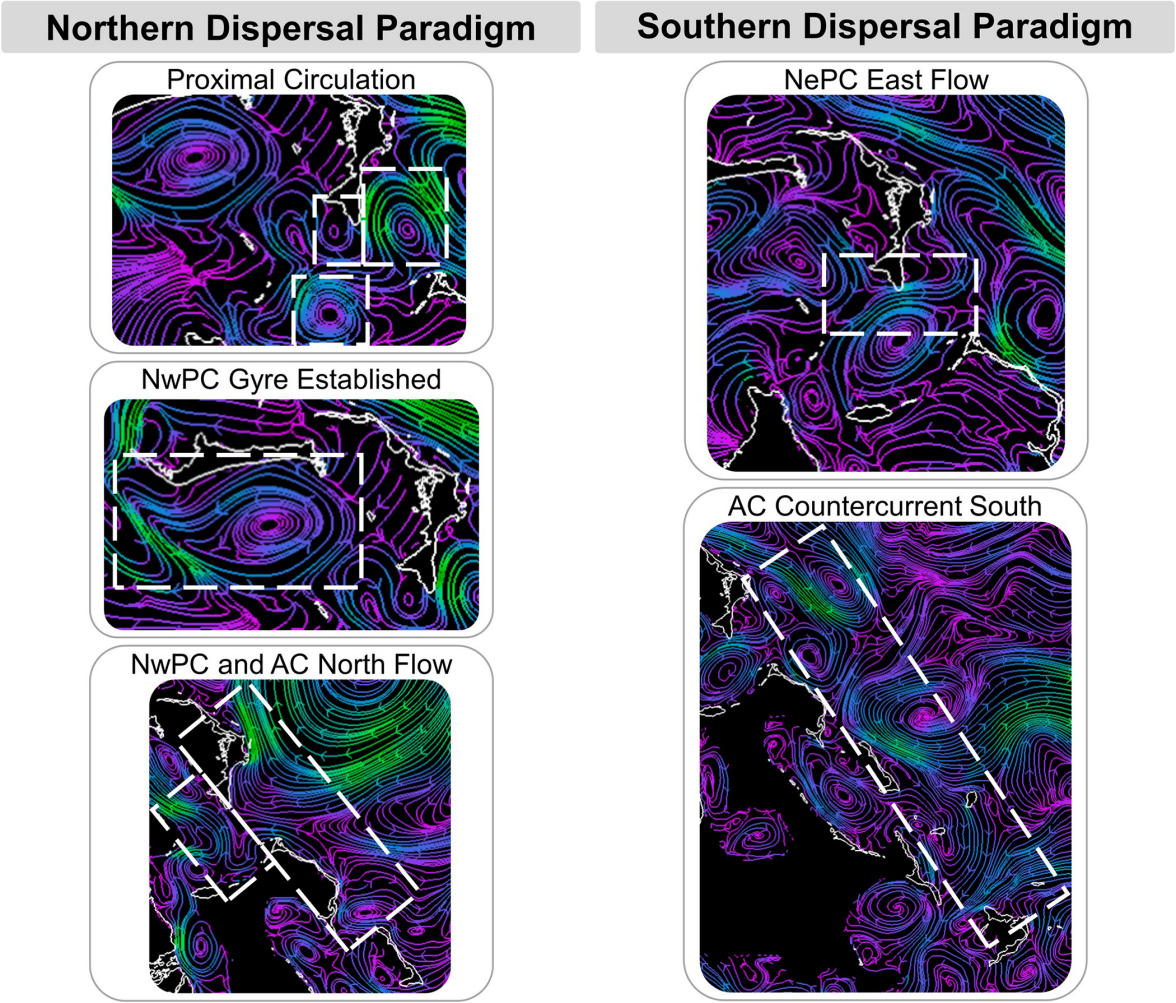
Dispersal paradigms. Two larval dispersal paradigms were evident across the LDMs: the prevailing Northern dominant and the anomalous Southern dominant. Each dispersal paradigm is promoted by a suite of hydrodynamic patterns depicted by flow vectors derived from the NCOM AmSeas hydrodynamic model. Hydrodynamic features are identified within the white hashed boxes. NwPC—Northwest Providence Channel, NePC—Northeast Providence Channel, AC—Antilles Current.

Larvae that did not experience mortality shortly after hatching circulated up to 71 DPS, allowing for inspection of unique and prevailing hydrodynamic conditions, including those associated with increased settlement success. Dispersal patterns across the years can be classified into two paradigms: Northern dominant and Southern dominant (Fig 9). Northern dominant dispersal is supported as the prevailing dispersal pattern given the consistency between the 2018 and 2019 LDMs. Furthermore, the underlying hydrodynamics follow known prevailing seasonal circulation patterns [98], and regional LDMs from other studies and species show dispersal patterns consistent with the Northern dominant paradigm [55, 99].

We did observe variability within the Northern dominant dispersal paradigm. In 2018, the presence of clustering mesoscale eddies within Northeast Providence Channel forced diffuse dispersal as portions of the larvae were divided among the periphery of a cyclonic eddy against the west facing shore of South Abaco and two anticyclonic eddies to the south and east of South Abaco. The anticyclonic eddy to the east subsequently passed a majority of the larvae to the north-flowing Antilles Current that flows along the eastern shores of Abaco, which was the main source of larval dispersal out of the Bahamian Archipelago and the model domain. Larvae within the southern anticyclonic eddy were moved south into TOTO and dispersed across New Providence and eastern Andros. The cyclonic eddy pushed larvae into the Northwest Providence Channel ephemeral gyre from the south, as the eddy and south flowing currents prevented larvae from dispersing westward immediately post spawn like what was observed in 2019. The 2019 spawn yielded the most successfully settled larvae and had the smaller of the two settlement footprints. A majority of the larvae were immediately drawn into the Northwest Providence Channel ephemeral gyre, mediated by the presence of an anticyclonic eddy positioned more proximally to the Little Bahama Bank between Abaco and Grand Bahama. Undisrupted inclusion into the Northwest Providence Channel gyre generated a slow and consistent dispersal of larvae to islands that interface with both Providence Channels. This also passed larvae westward into the Gulf Stream where larvae were lost from the northern domain.

The presence of the ephemeral gyre within Northwest Providence Channel undoubtedly is the most important circulation feature in determining the larval dispersal dynamics of bonefish sourced from the South Abaco spawn. Its presence provides a prevailing NW dispersal distribution and promotes larval retention within the northern extent of the Bahamian Archipelago. The regularity in which the gyre forms over winter [98] provides another predictable hydrodynamic feature in addition to the predictably stable thermocline that bonefish can rely upon to maximize larval survival [100]. Predictable and prevailing environments are central to the migratory spawning strategy, as significant energy investments are made prior to undergoing migrations to distant locations where conditions are unknown [11, 101].

The Southern dominant dispersal paradigm appears to be anomalous, only strictly occurring in 2013. Successful settlement was low given this paradigm, providing more support for the Northern dominant dispersal paradigm as the prevailing condition. However, the difference among the two paradigms might not be as large as presented here if not for the near total onshore mortality event in 2013. Conversely, there is the potential for complete mortality in the absence of prey availability if sufficient prey densities for larvae are reliant upon the presence of aggregating features, such as gyres and eddies [102], that were notably absent in the 2013 dispersal paths. The Southern dominant dispersal paradigm is characterized by an interaction with the Antilles Current, in particular the deep waters and counter current. The surface currents of the Antilles Current flow in a northward direction, while the counter current and deep waters flow south [98]. Evidence of a sustained southward flow along the eastern boundary of the Bahamian Archipelago can been seen across all years, with larval deposition varying between Eleuthera and Acklins. Strict adherence to the Southern dominant dispersal paradigm is anomalous, but the underlying currents do appear to be regularly involved in shaping the full dispersal footprint of the Northern dominant dispersal paradigm as well given the presence of southern dispersal along the eastern margin of the Bahamian Archipelago in 2018 and 2019.

These LDMs were uniquely constructed to incorporate new empirical information to simulate outcomes from observed spawning events. However, this effort was not the first to examine bonefish larval dispersal through LDMs, as Zeng et al. [55] did so to estimate regional connectivity of the Caribbean bonefish metapopulation. Comparisons between the two model structures and time periods allows for a more longitudinal assessment of the dispersal pathways, though parameterizations and metrics of success do vary considerably (Table 1). Zeng et al. [55] summarized dispersal as the final position for larvae at day 53, which is comparable to this study (Figs 2 and 3), as their final positions reported are not truly settlement. The density of final positions appears to capture the overall variability in dispersal observed in the LDMs within this study, with dispersal throughout the entire Bahamian Archipelago and Cuba; however, vagrants from the Bahamian Archipelago to the north are fewer in relative abundance to the rest of the domain and dispersal to Cuba is substantially greater. These differences can be attributed to contrasting programming in addition to hydrodynamic variation, inclusive of yearly, monthly, dispersal depth, and resolution differences. The density of final dispersal locations from 2009 to 2015 most closely resembles dispersal in 2018, with larval retention strongest within Northeast Providence Channel, though diffusive losses of larvae to the northeast most closely resemble 2019. It may be inferred from the combined timeseries of both Zeng et al. [55] and this study that the prevailing dispersal pattern is that of the Northern dominant paradigm and that larvae are most consistently distributed—independent of settlement—within Northeast Providence Channel.

**Table 1:** Comparison of LDM structure to Zeng et al. 2019.

LDM Parameterization	Abaco LDM	Zeng et al. (2019) LDM
Lagrangian Transport Model	Ichthyop	ROMS Coupled
Locations	Lombardo et al. 2020	Danylchuk et al. 2011
Larval Sources	3	1 (Site 2)
Resolution	1/36°	1/12°
Vertical Layers	40	36
Simulation Timespan	Nov–Jan 2013, 2018, 2019	1 Oct 2008–31 Dec 2015
Pelagic Larval Duration (Simulation Duration)	71 d	53 d
Larvae Released per Simulation	10000	100
Larvae Release Stain	3-dimensional, Thermocline Associated	Surface
Simulation Repetition Method	Same 4-dimensions	Each New and Full Moon October–May Each Year
Total Larvae	1000000	11800
Dispersal Depth Determination	Release depth + Advection	0.5 m
Diel Vertical Migration (DVM)	Depth of ≥ 2 m at 41 d	NA
Mortality Before Simulation Completed	Thermal Minima and Maxima Onshore/Beaching Beyond Model Domain	NA
Metric of Success	Reach Settlement Zone at 41–71 d	Gridded Location Densities

Comparison of our LDM parameterization with that of Zeng et al. [65]. Comparisons are only for LDM simulations with larvae sourced from South Abaco, The Bahamas.

#### Connectivity

The two larval dispersal paradigms yielded substantially different connectivity networks, with the Southern dominant dispersal paradigm in 2013 uniquely sourcing larvae southeast of San Salvador and beyond the southeastern extent of The Bahamas. In both paradigms, spawning events in Abaco have poor connectivity to islands southeast of New Providence. The Northern dominant paradigm sustained strong levels of self-recruitment and connectivity with islands north of New Providence, including Andros. Given that the hydrodynamic conditions in 2018 and 2019 align with prevailing conditions, the resultant settlement footprints can be inferred as the prevailing connectivity network for bonefish larvae sourced from Abaco. The prevailing connections are to Abaco, Grand Bahama, the Berry Islands, Eleuthera, Andros, and New Providence.

The viability of these connections is dependent upon the environments in which larvae settle, as habitats are not always suitable for a given life stage, disrupting the completion of connectivity (i.e., next generation spawning) [103]. We did not discriminate against shoreline composition, which can preclude larval settlement, especially for bonefish that require softly sloping sandy shorelines with low energy littoral zones [104]. Settlement positions within the LDMs are not necessarily definitive given that bonefish, like other fish [85, 105, 106], are likely to exhibit some capacity of spatiotemporal control during settlement to select for compatible nursery habitat. At Lee Stocking Island, bonefish recruitment showed strong periodicity with lunar or tidal cycles [53], suggesting that larvae can maintain their position until nighttime flood tide conditions are conducive to safe passage over the reefs [83] and into nearshore nursery habitats. The duration and conditions in which bonefish can maintain and delay settlement is unknown, and not a behavior that was possible to parameterize within the Ichthyop LDMs. We made an effort to capture the ability of positional maintenance within the settlement competency period by extending the settlement zones one to two grid cells beyond the shoreline (7.4 km maximum distance from shore); however, doing so may not entirely capture the behavior if larvae are able to maintain position before the 41 d lower limit of the settlement competency period. Given the unknown positional maintenance behaviors and the programmatic limitations in accounting for such behaviors, the assumption that successful settlement in the LDMs is equivalent to successful recruitment is required.

*Post hoc* ground truthing in future studies would allow for scenario-specific validation of LDM parameterization and results. Concurrent studies of genetic relatedness have proven useful in confirming connectivity and suggesting management strategies [70]. Assessments of genetic relatedness and connectivity independent of LDMs have also been applied to species in The Bahamas, including bonefish [107]. Douglas et al. [107] analyzed bonefish relatedness between Eleuthera and Grand Bahama, finding that bonefish on Grand Bahama’s ocean side (i.e., Northwest Providence Channel) and north exposed flats side are sink populations, as are bonefish at the southern extent of Eleuthera. These results and the results presented in seining efforts throughout The Bahamas can be used to provide context and ground truthing to the prevailing conditions observed in the LDMs within this study. Both Grand Bahama locations were strong recipients of larvae from the 2018 and 2019 spawns, while no larvae settled in the Eleuthera locations identified as sinks. While Douglas et al. [107] observed south-north source-sink dynamics between the Eleuthera and Grand Bahama, it is within reason that prevailing conditions promote similar dynamics between Abaco and Grand Bahama in support of the LDM observations. On Eleuthera, LDM larvae settled along the northern and eastern shoreline, while few larvae settle upon the leeward shores. Rigorous sampling for juvenile bonefish has only been conducted on Eleuthera, and efforts support prevailing transport to the windward side of Eleuthera where protected embayments contain nursery habitat [104]; however, CPUE of juvenile bonefish were comparatively higher at leeward stations within Rock Sound. Larvae in the 2018 suite of LDMs did reach Rock Sound in higher relative abundances than the windward embayments, though arrival to settlement habitat preceded the lower end of the settlement competency period (see Fig 2). Additional simulated sourcing of larvae to locations where juveniles have been found include Fresh Creek of Andros [108], Fish Creek and the Marls of Abaco (J. Lewis, pers. comm.), and August Creek and Pelican Creek of Grand Bahama (J. Lewis, pers. comm.).

### Larval development environment

Environments exert influence on physical performance characteristics during larval dispersal and development, as larvae exhibit phenotypic plasticity in traits such as growth rate, thermal tolerance, osmoregulation, and swimming performance [109]. Variability in phenotypic expression does influence the true measure of connectivity as phenotype-environment mismatches can reduce individual fitness, preventing the full cycle of connectivity where an individual further contributes offspring to the population [110]. Accordingly, assessing the larval development environments experienced by larvae as they disperse throughout The Bahamian Archipelago was important for gaining insight into significant variability that could confer advantages or deficiencies to larvae dispersed to certain islands.

The relative relationships of larval development environments for successfully settled larvae were largely consistent between 2018 and 2019, as both years followed the Northern dominant dispersal paradigm. However, the magnitude of differences was not equal between years, which can be attributed to variation in the hydrodynamics. Conversely, many comparisons indicated no differences between islands. Collectively, consistent relative relationships and null comparisons are indicative of prevailing conditions and consistency in the larval development environments across space and time.

The strongest contrasts in larval development environments per island were in the coefficients of dispersal and settlement DPS (Fig 7). The two metrics were not correlated, though both could potentially have strong influences on larval condition and success in adapting to littoral nursery habitats. Many of the islands in which larvae successfully settled had small coefficients of dispersal, implying that their dispersal pathways were heavily influenced by interacting with circulation features. Beyond their larval retention capacities, gyres and eddies create environments that have unique physical properties that strongly influence both biotic-abiotic and biotic-biotic interactions [111].

The primary circulation feature in the northern extent of the Bahamian Archipelago is the mesoscale gyre found within Northwest Providence Channel. The gyre forms in winter and is created by an influx of cool Atlantic water from the east through Northeast Providence Channel and warm Gulf Stream water from the west through Northwest Providence Channel [98]. Inputs from the Atlantic to the east and the Gulf Stream to the west are not equal or always consistent in their volume transport [98]. In 2013, this resulted in a deeper mixed layer depth. In 2018, a warm core anticyclonic eddy was present due to elevated velocity intrusion of cold water from Little Bahama Bank [98]. And in 2019, a slower velocity warm core anticyclonic eddy was present (S2E-S2G Fig). Gyre presence under prevailing conditions places larvae into highly productive conditions, where both upwelling from cyclonic eddies within the > 1000 m deep Providence Channels and the downwelling anticyclonic gyre can supply nutrients for phytoplankton and zooplankton to thrive in an otherwise nutrient deficient environment [112, 113].

The stable thermal stratification of the region in winter and eddy presence appears to be a larval development environment that is tightly coupled to bonefish evolutionary history as congeners in the Gulf of California have been found in their highest abundances within the same environment [21]. Accordingly, larvae with higher coefficients of dispersal may have increased access to prey, and subsequently have improved body condition and higher survival upon settlement. The positive relationship between body condition and settlement survival has been documented in reef fish [114], and body condition can even offset deficiencies in competitive ability brought on by late settlement (i.e., priority effects) [115–117]. Bonefish larvae settling in The Bahamas are likely to be less dependent upon the compensatory competitive advantage of settling with higher body condition than reef fish due to nursery habitat composition and obligations. Bonefish exploit unstructured littoral nursery habitats that are not constrained by competitive or predatory priority effects, as such habitats in The Bahamas are plentiful and the success of their recruitment appears to be facilitated by inter-species schooling with gregarious small-bodied fish [118, 119]. More research is needed to understand how body condition in settlement stage bonefish effects recruitment.

Differences between the other successfully settled larval development environment measures—depth released, mean dispersal depth, and mean temperature—were much smaller and more inconsistent in their relative relationships (Figs 7 and 8). However, consistency across islands can support the presence of predictable and stable environments that are vital to larval survival. The peak distributions of mean dispersal depths were shallower than release depths across all islands and were closely associated with the thermocline. Larvae are universally found more frequently associated with stratified oceanographic features and have both biological and behavioral means to maintain close association [112]. The boundary layer within the stratification feature provides insular protection from the larger stochastic processes that occur within the larger water masses, providing reduced turbulence and flow velocities [120] over large amplitude orbital motions [100], more predictable dispersal [121], and prey availability [112]. The strong association of the deep chlorophyll maximum and the thermocline within the eastern Caribbean exemplifies the nutritional benefits of thermocline associations for larval fish within The Bahamas [122]. The buoyancy of bonefish eggs and larvae [123, 124] are most likely to have imparted the consistent relationship between the thermocline and mean dispersal depth. An instantaneous buoyancy rate of 8.5 m·d^-1^ at 0 h post-hatch (25 h post-fertilization) was calculated using the standard Stokes’ Law following the methods in McDonnell and Buesseler [125] with a larval density of 1.02 × 10^−5^ kg·m^-3^, head diameter of 2 × 10^−4^ m, a length of 0.005 m [123], a dynamic viscosity of 0.959 kg·m^-1·s^ [126], and a water density of 1024.4 kg·m^-3^. The buoyancy rate allowed for larvae to rise towards the thermocline, and the rate of decreasing buoyancy over development reached a nearly neutral specific gravity in close temporal proximity to the larvae reaching the thermocline.

Temperature also significantly impacts larval development, growth, and survival [109]. While temperature effects on bonefish larvae are unknown, Japanese eels in captivity experienced higher hatching and survival rates, as well as faster hatching, prefeeding growth, and onset of feeding in waters progressively warmer from 19–28°C [127]. Similar results were observed in tropical reef fish [128]. Consistency in the positive relationship between temperature and development suggests warmer conditions would benefit bonefish larval development; however, the lower and upper range of beneficial temperatures cannot be assumed. Future research into how temperature impacts bonefish larval development is needed in order to fully assess the implications of variable temperatures during dispersal.

### Future management and bonefish research suggestions

Zeng et al. [55] concluded that management plans effectuated at scales pertaining to isolated stocks were not sufficient to effectively conserve bonefish populations within the Caribbean. The same issue of scaling can be applied at an intra-jurisdictional scale within The Bahamas. While the harvest of bonefish is restricted to subsistence fishing within The Bahamas [33], loss of PSA and nursery habitats remains a present threat to bonefish populations [38]. The Bahamas has established 77 marine protected areas (MPAs), marine managed areas (MMAs), fisheries reserves, ecological reserves, and national parks that protect marine and shore habitats [129], with bonefish as a focal species for habitat protection in some of these areas. Tagging studies focusing on the movements of bonefish from home flats to PSA locations [14, 48] helped to inform park inclusion of bonefish habitats. Data on nursery habitats and settlement patterns did not exist for consultation and incorporation into spatial management plans. The results of our study show an alignment of core settlement areas and park protected areas throughout Abaco, Grand Bahama, the Berry Islands, and Northern Andros. Within these locations, protections from development and water quality degradation should be sufficient to protect nursery habitats and larvae sourced from spawning events in Abaco. Our models did not discern potential bonefish nursery habitats through remote sensing or *in situ* assessments. All shorelines were included as potential settlement zones, as the capabilities of bonefish larvae to move through habitats is unknown. Furthermore, methods to delineate bonefish nursery habitats are still being refined and are labor intensive [54, 104]. Accordingly, suggestions to include other locations with high concentrations of larval influx to the Bahamas’ parks and spatial protections programs requires assessments of habitats to delineate nursery status. We suggest further examination and consideration of potential nursery habitats in the core settlement areas of Moore’s Island Abaco (northwest of the spawning locations), the central most eastern islands of the Berry Islands, and North Eleuthera that would result in the creation of new parks. Expanding and conjoining of Marls of Abaco National Park (west central Abaco) to Cross Harbor National Park (South Abaco) would encompass the 23 km of catchment to the north that contributes to the South Abaco PSA [14], and thus would be inclusive of self-recruiting larvae. Larval supply was strong along the northern shore of Grand Bahama, and westward expansion of North Shore/The Gap National Park (north central Grand Bahama) to conjoin with West End MPA (northwest Grand Bahama) would protect recruitment to this region. A core settlement area was observed upon the northern tip of Andros, where Joulter Cays National Park encompasses the sand flats habitats that North Andros bonefish occupy. However, Joulter Cays National Park does not extend to the creeks and marls between the sand flats and mainland of North Andros—habitats that are likely to support juvenile bonefish recruitment. Expansion of Joulter Cays National Park southward to include the creek and marl habitats is recommended. Core settlement areas were also observed in East New Providence and West Grand Bahama; however, both regions are heavily developed by nearby Nassau and Freeport, which have already contributed to the destruction of nursery habitats and low bonefish densities found in those regions.

As new biological information is discovered on the spawning and early life history of bonefish, the construct of bonefish LDMs should be further refined to increase accuracy in representing the dispersal of larvae. We suggest future research subjects that will contribute to bonefish LDMs. First, prioritize describing ontogenetic changes in larval swimming capabilities, in both the pelagic and nearshore environments, since larval behavior can significantly influence dispersal and connectivity [76]. Second, evaluate the relationship between bonefish spawning site selection and physical ocean conditions. Additional bonefish spawning tracks should be conducted while implementing the use of a CTD with a depth rating > 100 m to capture the bottom extent of the offshore migration in instances where fish move below 100 m. The required equipment will be a logistical constraint, as the CTD and retrieval equipment needed to sample such depths increase in size and mass. Finally, future bonefish LDM studies should incorporate ground truthing using juvenile habitat surveys [see 105] and/or larval collections [see 53] and evaluate genetic relatedness. Collectively, these research objectives will fill knowledge gaps in bonefish early life history that have persisted for decades, and ultimately help to inform conservation measures across the Greater Caribbean.

## Supporting information

S1 File Ichthyop configuration XML file. (XML)

S2 File Relating *Albula vulpes* density development to *Anguilla japonica*. (DOCX)

S1 Fig Comparison of NCOM AmSeas hydrography data to *in situ* Castaway CTD data. (DOCX)

S2 Fig Larval dispersal footprints, hydrography, and successful settlement across depth bins. (DOCX)

S1 Table Larval development environment by settled island Kruskall-Wallis and Dunn’s Test results. (DOCX)

## References

[1] Cowen RK, and Sponaugle S. Larval dispersal and marine population connectivity. Annual Review of Marine Science. 2009; 1:443–466. doi: 10.1146/annurev.marine.010908.163757 21141044

[2] Gotelli NJ. Metapopulation models: the rescue effect, the propagule rain, and the core-satellite hypothesis. The American Naturalist. 1991; 138(3):768–776.

[3] Runge JP, Runge MC, and Nichols JD. The role of local populations within a landscape context: defining and classifying sources and sinks. The American Naturalist. 2006; 167(6):925–938. doi: 10.1086/503531 16615034

[4] Hastings A, and Botsford LW. Persistence of spatial populations depends on returning home. Proceedings of the National Academy of Sciences. 2006; 103(15):6067–6072. doi: 10.1073/pnas.0506651103 16608913 PMC1458697

[5] Schindler DE, Hilborn R, Chasco B, Boatright CP, Quinn TP, Rogers LA, et al. Population diversity and the portfolio effect in an exploited species. Nature. 2010; 465(7298):609–612. doi: 10.1038/nature09060 20520713

[6] Carson HS, Cook GS, López-Duarte PC, and Levin LA. Evaluating the importance of demographic connectivity in a marine metapopulation. Ecology. 2011; 92(10):1972–1984. doi: 10.1890/11-0488.1 22073788

[7] Kerr LA, Hintzen NT, Cadrin SX, Clausen LW, Dickey-Collas M, Goethel DR, et al. Lessons learned from practical approaches to reconcile mismatches between biological population structure and stock units of marine fish. ICES Journal of Marine Science. 2017; 74(6):1708–1722. doi: 10.1093/icesjms/fsw188

[8] Hilborn R, Stokes K, Maguire JJ, Smith T, Botsford LW, Mangel M, et al. When can marine reserves improve fisheries management? Ocean & Coastal Management. 2004; 47(3–4):197–205. doi: 10.1016/j.ocecoaman.2004.04.001

[9] Planes S, Jones GP, and Thorrold SR. Larval dispersal connects fish populations in a network of marine protected areas. Proceedings of the National Academy of Sciences. 2009; 106(14):5693–5697. doi: 10.1073/pnas.0808007106 19307588 PMC2659712

[10] Adams AJ, Rehage JS, and Cooke SJ. A multi-methods approach is essential for effective conservation and management of recreational flats fisheries. Environmental Biology of Fishes. 2019; 102(2):105–115. doi: 10.1007/s10641-018-0840-1

[11] Domeier ML, and Colin PL. Tropical reef fish spawning aggregations: defined and reviewed. Bulletin of Marine Science. 1997; 60(3):698–726.

[12] Ciannelli L, Bailey K, Olsen EM. Evolutionary and ecological constraints of fish spawning habitats. ICES Journal of Marine Science. 2015; 72(2):285–296. doi: 10.1093/icesjms/fsu145

[13] Secor DH, Kerr LA, and Cadrin SX. Connectivity effects on productivity, stability, and persistence in a herring metapopulation model. ICES Journal of Marine Science 2009; 66(8):1726–1732. doi: 10.1093/icesjms/fsp154

[14] Boucek RE, Lewis JP, Stewart BD, Jud ZR, Carey E, and Adams AJ. Measuring site fidelity and homesite-to-pre-spawning site connectivity of bonefish (*Albula vulpes*): using mark-recapture to inform habitat conservation. Environmental Biology of Fishes. 2019; 102(2):185–195. doi: 10.1007/s10641-018-0827-y

[15] Sadovy De Mitcheson Y, Cornish A, Domeier M, Colin PL, Russell M, and Lindeman KC. A global baseline for spawning aggregations of reef fishes. Conservation Biology. 2008; 22(5):1233–1244. doi: 10.1111/j.1523-1739.2008.01020.x 18717693

[16] Frisk MG, Jordaan A, and Miller TJ. Moving beyond the current paradigm in marine population connectivity: are adults the missing link?. Fish and Fisheries. 2014; 15(2):242–254. doi: 10.1111/faf.12014

[17] D’Aloia CC, Bogdanowicz SM, Francis RK, Majoris JE, Harrison RG, and Buston PM. Patterns, causes, and consequences of marine larval dispersal. Proceedings of the National Academy of Sciences. 2015; 112(45):13940–13945. doi: 10.1073/pnas.1513754112 26508628 PMC4653176

[18] Treml EA, Ford JR, Black KP, and Swearer SE. Identifying the key biophysical drivers, connectivity outcomes, and metapopulation consequences of larval dispersal in the sea. Movement Ecology. 2015; 3(1):1–16. doi: 10.1186/s40462-015-0045-6 26180636 PMC4502943

[19] Pineda J, Hare JA, and Sponaugle S. Larval transport and dispersal in the coastal ocean and consequences for population connectivity. Oceanography. 2007; 20:22–39. doi: 10.5670/oceanog.2007.27

[20] Sánchez-Velasco L, Jiménez-Rosenberg SPA, and Lavín MF. Vertical distribution of fish larvae and its relation with water column structure in the SW of the Gulf of California. Pacific Science. 2007; 61(4):533–548. doi: 10.2984/1534-6188(2007)61[533:VDOFLA]2.0.CO;2

[21] Sánchez-Velasco L, Lavín MF, Jimėnez-Rosenberg SPA, Godínez VM, Santamaría-del-Angel E, and Hernández-Becerril DU. Three-dimensional distribution of fish larvae in a cyclonic eddy in the Gulf of California during the summer. Deep Sea Research Part I. 2013; 75:39–51. https://doi.org/10.1016/j.dsr.2013.01.009.

[22] Habtes S, Muller‐Karger FE, Roffer MA, Lamkin JT, and Muhling BA. A comparison of sampling methods for larvae of medium and large epipelagic fish species during spring SEAMAP ichthyoplankton surveys in the Gulf of Mexico. Limnology and Oceanography: Methods. 2104; 12(2):86–101. doi: 10.4319/lom.2014.12.86

[23] Swearer SE, Caselle JE, Lea DW, and Warner RR. Larval retention and recruitment in an island population of a coral-reef fish. Nature. 1999; 402(6763):799–802. doi: 10.1038/45533

[24] Swearer SE, Treml EA, and Shima J. A review of biophysical models of marine larval dispersal. In Hawkins SJ, Allcock AL, Bates AE, Firth LB, Smith IP, Swearer SE, et al. (eds) Oceanography and Marine Biology. CRC Press, Taylor & Francis Group, Boca Raton; 2019. pp. 325–356.

[25] Haidvogel DB, Arango H, Budgell WP, Cornuelle BD, Curchitser E, Di Lorenzo E, et al. Ocean forecasting in terrain-following coordinates: Formulation and skill assessment of the Regional Ocean Modeling System. Journal of Computational Physics. 2008; 227(7):3595–3624. doi: 10.1016/j.jcp.2007.06.016

[26] Marsaleix P, Auclair F, and Estournel C. Considerations on Open Boundary Conditions for Regional and Coastal Ocean Models. Journal of Atmospheric and Oceanic Technology. 2006; 23:1604–1613. doi: 10.1175/JTECH1930.1

[27] Barron CN, Kara A, Martin P, Rhodes R, and Smedstad L. Formulation, implementation and examination of vertical coordinate choices in the Global Navy Coastal Ocean Model (NCOM). Ocean Model. 2006; 11:347–375. doi: 10.1016/j.ocemod.2005.01.004

[28] North EW, Hood RR, Chao SY, and Sanford LP. Using a random displacement model to simulate turbulent particle motion in a baroclinic frontal zone: A new implementation scheme and model performance tests. Journal of Marine Systems. 2006; 60:365–380. doi: 10.1016/j.jmarsys.2005.08.003

[29] Paris CB, Helgers J, Van Sebille E, and Srinivasan A. Connectivity Modeling System: A probabilistic modeling tool for the multi-scale tracking of biotic and abiotic variability in the ocean. Environmental Modelling & Software. 2013; 42:47–54. doi: 10.1016/j.envsoft.2012.12.006

[30] Lange M, and van Sebille E. Parcels v0.9: prototyping a Lagrangian ocean analysis framework for the petascale age. Geoscientific Model Development. 2017; 10(11):4175–4186. doi: 10.5194/gmd-10-4175-2017

[31] Delandmeter P, and van Sebille E. The Parcels v2.0 Lagrangian framework: new field interpolation schemes. Geoscientific Model Development. 2019; 12(8):3571–3584. doi: 10.5194/gmd-12-3571-2019

[32] Lett C, Verley P, Mullon C, Parada C, Brochier T, Penven P, et al. A Lagrangian tool for modelling ichthyoplankton dynamics. Environmental Modelling & Software. 2008; 23(9):1210–1214. doi: 10.1016/j.envsoft.2008.02.005

[33] Danylchuk A, Danylchuk S, Philipp DP, Goldberg TL, Cooke SJ, and Koppelman J. Ecology and management of bonefish (*Albula* spp.) in the Bahamian Archipelago. In: Ault JS (ed) Biology and Management of the World Tarpon and Bonefish Fisheries. CRC Press, Taylor & Francis Group, Boca Raton. 2007; pp. 99–112.

[34] Adams AJ. Guidelines for evaluating the suitability of catch and release fisheries: lessons learned from Caribbean flats fisheries. Fisheries Research. 2017; 186:672–680. doi: 10.1016/j.fishres.2016.09.027

[35] Fedler T. Economic impact of the Florida Keys flats fishery. The Bonefish and Tarpon Trust. Gainesville, Florida. 2013; [Online] Available from: https://www.bonefishtarpontrust.org/downloads/research-reports/stories/BTT%20-%20Keys%20Economic%20Report.pdf. [Accessed August 2018].

[36] Fedler AJ. 2013 economic impact of flats fishing in Belize. 2014; [Online] Available from: https://www.bonefishtarpontrust.org/downloads/research-reports/stories/2013-belize-economic-study.pdf. [Accessed August 2020].

[37] Fedler T. The 2018 economic impact of flats fishing in the Bahamas. Report to the Bonefish and Tarpon Trust. 2019; [Online] Available from: https://www.bonefishtarpontrust.org/downloads/research-reports/stories/bahamas-flats-economic-impact-report.pdf. [Accessed August 2020].

[38] Adams AJ, Horodysky AZ, McBride RS, Guindon K, Shenker JM, MacDonald TC, et al. Global conservation status and research needs for tarpons (Megalopidae), ladyfishes (Elopidae) and bonefishes (Albulidae). Fish and Fisheries. 2014; 15(2), 280–311. 10.1111/faf.12017

[39] Santos RO, Rehage JS, Kroloff EKN., Heinen JE, and Adams AJ. Combining data sources to elucidate spatial patterns in recreational catch and effort: fisheries-dependent data and local ecological knowledge applied to the South Florida bonefish fishery. Environmental Biology of Fishes. 2019; 102:299–317. doi: 10.1007/s10641-018-0828-x

[40] Brownscombe JW, Griffin LP, Gagne TO, Haak CR, Cooke SJ, Finn JT, et al. Environmental drivers of habitat use by a marine fish on a heterogeneous and dynamic reef flat. Marine Biology. 2019; 166(2):18. doi: 10.1007/s00227-018-3464-2

[41] Cooke SJ, and Philipp DP. Behavior and mortality of caught-and-released bonefish (*Albula* spp.) in Bahamian waters with implications for a sustainable recreational fishery. Biological Conservation. 2004; 118(5):599–607. doi: 10.1016/j.biocon.2003.10.009

[42] Danylchuk SE, Danylchuk AJ, Cooke SJ, Goldberg TL, Koppelman J, and Philipp DP. Effects of recreational angling on the post-release behavior and predation of bonefish (*Albula* vulpes): the role of equilibrium status at the time of release. Journal of Experimental Marine Biology and Ecology. 2007; 346:127–133. doi: 10.1016/j.jembe.2007.03.008

[43] Brownscombe JW, Danylchuk AJ, Chapman JM, Gutowsky LF, and Cooke SJ Best practices for catch-and-release recreational fisheries–angling tools and tactics. Fisheries Research. 2017; 186:693–705. doi: 10.1016/j.fishres.2016.04.018

[44] Danylchuk AJ, Cooke SJ, Goldberg TL, Suski CD, Murchie KJ, Danylchuk SE, et al. Aggregations and offshore movements as indicators of spawning activity of bonefish (*Albula vulpes*) in the Bahamas. Marine Biology. 2011; 158(9):1981–1999. doi: 10.1007/s00227-011-1707-6

[45] Danylchuk AJ, Lewis JP, Jud ZR, Shenker JM, and Adams AJ. Behavioral observations of bonefish (*Albula vulpes*) during prespawning aggregations in The Bahamas: clues to identifying spawning sites that can drive broader conservation efforts. Environmental Biology of Fishes. 2019; 102(1):175–184. doi: 10.1007/s10641-018-0830-3

[46] Adams AJ, Shenker JM, Jud Z, Lewis J, Carey E, and Danylchuk AJ. Identifying pre-spawning aggregation sites for bonefish (*Albula vulpes*) to inform habitat protection and species conservation. Environmental Biology of Fishes. 2019; 102:159–173. doi: 10.1007/s10641-018-0802-7

[47] Perez AU, Schmitter-Soto JJ, Adams AJ, and Heyman WD. Connectivity mediated by seasonal bonefish (*Albula vulpes*) migration between the Caribbean Sea and a tropical estuary of Belize and Mexico. Environmental Biology of Fishes. 2019; 102:197–207. doi: 10.1007/s10641-018-0834-z

[48] Adams AJ, Lewis JP, Kroetz AM, and Grubbs RD. Bonefish (*Albula vulpes*) home range to spawning site linkages support a marine protected area designation. Aquatic Conservation: Marine and Freshwater Ecosystems. 2021; 31(6):1346–1353. doi: 10.1002/aqc.3534

[49] Lombardo SM, Adams AJ, Danylchuk AJ, Luck CA, and Ajemian MJ. Novel deep-water spawning patterns of bonefish (*Albula vulpes*), a shallow water fish. Marine Biology. 2020; 167(12):1–11. doi: 10.1007/s00227-020-03799-3

[50] Adams AJ, and Cooke SJ. Advancing the science and management of flats fisheries for bonefish, tarpon, and permit. Environmental Biology of Fishes. 2015; 98(11):2123–2131. doi: 10.1007/s10641-015-0446-9

[51] Filous A, Lennox RJ, Raveino R, Friedlander AM, Clua EE, Cooke SJ, et al. The spawning migrations of an exploited Albulid in the tropical Pacific: implications for conservation and community-based management. Environmental Biology of Fishes. 2020; 103(9):1013–1031. doi: 10.1007/s10641-020-00996-3

[52] Filous A, Lennox RJ, Beaury JP, Bagnis H, Mchugh M, Friedlander AM, et al. Fisheries science and marine education catalyze the renaissance of traditional management (rahui) to improve an artisanal fishery in French Polynesia. Marine Policy. 2021; 123:104291. doi: 10.1016/j.marpol.2020.104291

[53] Mojica R Jr., Shenker JM, Harnden CW, and Wagner DE. Recruitment of bonefish, *Albula vulpes*, around Lee Stocking Island, Bahamas. Fisheries Bulletin-NOAA. 1995; 93:666–674.

[54] Haak CR, Power M, Cowles GW, and Danylchuk AJ. Hydrodynamic and isotopic niche differentiation between juveniles of two sympatric cryptic bonefishes, *Albula vulpes* and *Albula goreensis*. Environmental Biology of Fishes. 2019; 102(2):129–145. doi: 10.1007/s10641-018-0810-7

[55] Zeng X, Adams A, Roffer M, and He R. Potential connectivity among spatially distinct management zones for Bonefish (*Albula vulpes*) via larval dispersal. Environmental Biology of Fishes. 2019. 102(2):233–252. doi: 10.1007/s10641-018-0826-z

[56] Heyman WD, Kjerfve B, Graham RT, Rhodes, and Garbutt L. Spawning aggregations of *Lutjanus cyanopterus* (Cuvier) on the Belize Barrier Reef over a 6 year period. Journal of Fish Biology. 2005; 67(1):83–101. doi: 10.1111/j.0022-1112.2005.00714.x

[57] Nero RW, Cook M, and Coleman AT. Using an ocean model to predict likely drift tracks of sea turtle carcasses in the north central Gulf of Mexico. Endangered Species Research. 2013; 21:191–203. doi: 10.3354/esr00516

[58] O’Conner BS, Muller-Karger FE, and Redwood RW. The role of Mississippi River discharge in offshore phytoplankton blooming in the northeastern Gulf of Mexico during August 2010. Remote Sensing of Environment. 2016; 173:133–144. doi: 10.1016/j.rse.2015.11.004

[59] Garavelli L, Studivan MS, Voss JD, Kuba A, Figueiredo J, and Chérubin LM Assessment of mesophotic coral ecosystem connectivity for proposed expansion of a marine sanctuary in the northwest Gulf of Mexico: larval dynamics. Frontiers in Marine Science. 2018; 5:174. doi: 10.3389/fmars.2018.00174

[60] Zaron ED, Fitzpatrick PJ, Cross SL, Harding JM, Bub FL, Wiggert JD, et al. Initial evaluations of a Gulf of Mexico/Caribbean ocean forecast system in the context of the Deepwater Horizon disaster. Frontiers in Earth Science. 2015; 9:605–636. doi: 10.1007/s11707-014-0508-x

[61] Cummings JA. Ocean Data Quality Control. In: Schiller A., and Brassington G. B. (eds), Operational Oceanography in the 21st Century. Springer, New York. 2011; pp. 91–121.

[62] Chassignet EP, Hurlburt HE, Smedstad OM, Halliwell GR, Hogan PJ, Wallcraft AJ, et al. The HYCOM (hybrid coordinate ocean model) data assimilative system. Journal of Marine Systems. 2007; 65(1–4):60–83. doi: 10.1016/j.jmarsys.2005.09.016

[63] Hogan TF, Liu M, Ridout JA, Peng MS, Whitcomb TR, Ruston BC, et al. The Navy Global Environmental Model. Oceanography. 2014; 27:116–125. https://www.jstor.org/stable/24862194.

[64] Egbert GD, and Erofeeva SY. Efficient inverse modeling of barotropic ocean tides, Journal of Atmospheric and Ocean Technology. 2002; 19(2):183–204. doi: 10.1175/1520-0426(2002)019&lt;0183:EIMOBO&gt;2.0.CO;2

[65] Zaron ED. Predictability of non-phase-locked baroclinic tides in the Caribbean Sea. Ocean Science. 2019; 15(5):1287–1305. doi: 10.5194/os-15-1287-2019

[66] Penven P, Marchesiello P, Debreu L, and Lefevre J. Software tools for pre-and post-processing of oceanic regional simulations. Environmental Modelling & Software. 2008; 23(5):660–662. doi: 10.1016/j.envsoft.2007.07.004

[67] Amante C. ETOPO1 1 arc-minute global relief model: procedures, data sources and analysis. Boulder, CO. U.S. Dept. of Commerce, National Oceanic and Atmospheric Administration, National Environmental Satellite, Data, and Information Service, National Geophysical Data Center, Marine Geology and Geophysics Division. 2009; doi: 10.7289/V5C8276M

[68] Wessel P, and Smith WHF. A Global Self-consistent, Hierarchical, High-resolution Shoreline Database. Journal of Geophysical Research. 1996; 101(B4):8741–8743. doi: 10.1029/96JB00104

[69] Chérubin LM, and Garavelli L. Eastern Caribbean circulation and island mass effect on St. Croix, US Virgin Islands: a mechanism for relatively consistent recruitment patterns. PLOS One. 2016; 11(3):p.e0150409. doi: 10.1371/journal.pone.0150409 26942575 PMC4778804

[70] Segura-García I, Garavelli L, Tringali M, Matthews T, Chérubin LM, Hunt J, et al. Reconstruction of larval origins based on genetic relatedness and biophysical modeling. Scientific Reports. 2019; 9(1):1–9. doi: 10.1038/s41598-019-43435-9 31068625 PMC6506592

[71] Courant R, Friedrichs K, and Lewy H. Über die partiellen Differenzengleichungen der mathematischen Physik. Mathematische Annalen. 1928; 100(1):32–74. doi: 10.1007/BF01448839

[72] Pelíz A, Marchesiello P, Dubert J, Marta-Almeida M, Roy C, and Queiroga H. A study of crab larvae dispersal on the Western Iberian Shelf: physical processes. Journal of Marine Systems. 2007; 68:215–236. doi: 10.1016/j.jmarsys.2006.11.007

[73] Okubo A. Oceanic diffusion diagrams. Deep-Sea Research and Oceanographic Abstracts. 1971; 18(8):789–802. doi: 10.1016/0011-7471(71)90046-5

[74] Visser AW. Using random walk models to simulate the vertical distribution of particles in a turbulent water column. Marine Ecology Progress Series. 1997; 158:275e281. doi: 10.3354/meps158275

[75] Simons RD, Siegel DA, and Brown KS. Model sensitivity and robustness in the estimation of larval transport: a study of particle tracking parameters. Journal of Marine Systems. 2013; 119:19–29. doi: 10.1016/j.jmarsys.2013.03.004

[76] Paris CB, Chérubin LM, and Cowen RK. Surfing, spinning, or diving from reef to reef: effects on population connectivity. Marine Ecology Progress Series. 2007; 347:285–300. doi: 10.3354/meps06985

[77] Halstead WR, Mejri S, Cianciotto AC, Wills PS, Van Leeuwen TE, Adams AJ, et al. Induced spawning and embryonic and early larval development of bonefish (*Albula vulpes*). Journal of Fish Biology. 2020; 96(3):825–830. doi: 10.1111/jfb.14250 31900926

[78] Miller MJ. Ecology of anguilliform leptocephali: remarkable transparent fish larvae of the ocean surface layer. Aqua-BioScience Monographs. 2009; 2(4):1–94.

[79] Tsukamoto K, Yamada Y, Okamura A, Kaneko T, Tanaka H, Miller MJ, et al. Positive buoyancy in eel leptocephali: an adaptation for life in the ocean surface layer. Marine Biology. 2009; 156(5):835–846. doi: 10.1007/s00227-008-1123-8

[80] Pfeiler E. Inshore migration, seasonal distribution and sizes of larval bonefish, Albula, in the Gulf of California. Environmental Biology of Fishes. 1984; 10(1):117–122. doi: 10.1007/BF00001668

[81] Pfeiler E, Mendoza MA, and Manrique FA. Premetamorphic bonefish (Albula sp.) leptocephali from the Gulf of California with comments on life history. Environmental Biology of Fishes. 1988; 21(4):241–249. doi: 10.1007/BF00000373

[82] Gilmore RG, Bullock LH, and Berry FH. Hypothermal mortality in marine fishes of South-Central Florida January, 1977. Northeast Gulf Science. 1978; 2(2):77–97. [Online] Available from: http://fau.digital.flvc.org/islandora/object/fau%3A6353. [Accessed December 2019].

[83] Hamner WM, Jones MS, Carleton JH, Hauri IR, and Williams DM. Zooplankton, planktivorous fish, and water currents on a windward reef face: Great Barrier Reef, Australia. Bulletin of Marine Science. 1988; 42(3):459–479.

[84] Peck MA, Huebert KB, and Llopiz JK. Intrinsic and extrinsic factors driving match-mismatch dynamics during the early life history of marine fishes. In: Advances in Ecological Research. 2012; Vol. 47: pp. 177–302. Academic Press. Orlando. doi: 10.1016/B978-0-12-398315-2.00003-X

[85] Shima JS, Noonburg EG, Swearer SE, Alonzo SH, and Osenberg CW. Born at the right time? A conceptual framework linking reproduction, development, and settlement in reef fish. Ecology. 2018; 99(1):116–126. doi: 10.1002/ecy.2048 29032595

[86] Ripley B, Venables B, Bates DM, Hornik K, Gebhardt A, Firth D, et al. Package ‘mass’. Cran r. 2013;538:113–20.

[87] Hedges LV. Distribution theory for Glass’s estimator of effect size and related estimators. Journal of Educational Statistics. 1981; 6(2):107–28. doi: 10.3102%2F10769986006002107.

[88] Hedges LV, and Olkin I. Statistical methods for meta-analysis. Academic Press, Inc. Orlando. 1985.

[89] Cohen J. A power primer. Psychological Bulletin. 1992; 112(1):155–159. doi: 10.1037//0033-2909.112.1.155 19565683

[90] Dinno A. dunn.test: Dunn’s Test of Multiple Comparisons Using Rank Sums. R package version 1.3.5. 2017; https://CRAN.R-project.org/package=dunn.test.

[91] Torchiano M. effsize: Efficient Effect Size Computation. R package version 0.8.1. 2020; doi: 10.5281/zenodo.1480624

[92] Pfeiler E. Salinity tolerance of leptocephalous larvae and juveniles of the bonefish (Albulidae: Albula) from the Gulf of California. Journal of Experimental Marine Biology and Ecology. 1981; 52(1):37–45. doi: 10.1016/0022-0981(81)90169-6

[93] García A, Alemany F, Velez-Belchí P, López Jurado JL, Cortés D, De la Serna JM, et al. Characterization of the bluefin tuna spawning habitat off the Balearic Archipelago in relation to key hydrographic features and associated environmental conditions. Collective Volume of Scientific Papers ICCAT. 2005; 58(2):535–49.

[94] Hátún H, Payne MR, and Jacobsen JA. The North Atlantic subpolar gyre regulates the spawning distribution of blue whiting (*Micromesistius poutassou*). Canadian Journal of Fisheries and Aquatic Sciences. 2009; 66(5):759–770. doi: 10.1139/F09-037

[95] Schabetsberger R, Miller MJ, Olmo GD, Kaiser R, Økland F, Watanabe S, et al. Hydrographic features of anguillid spawning areas: potential signposts for migrating eels. Marine Ecology Progress Series. 2016; 554:141–55. doi: 10.3354/meps11824 33184524 PMC7116345

[96] Shropshire TA, Morey SL, Chassignet EP, Coles VJ, Karnauskas M, Malca E, et al. Trade-offs between risks of predation and starvation in larvae make the shelf break an optimal spawning location for Atlantic Bluefin tuna. Journal of Plankton Research. 2021; fbab041. doi: 10.1093/plankt/fbab041 36045951 PMC9424715

[97] Liu Y, Lee SK, Enfield DB, Muhling BA, Lamkin JT, Muller-Karger FE, et al. Potential impact of climate change on the Intra-Americas Sea: Part-1. A dynamic downscaling of the CMIP5 model projections. Journal of Marine Systems. 2015; 148:56–69. doi: 10.1016/j.jmarsys.2015.01.007

[98] Chérubin LM. High-resolution simulation of the circulation in the Bahamas and Turks and Caicos Archipelagos. Progress in Oceanography. 2014; 127:21–46. doi: 10.1016/j.pocean.2014.05.006

[99] Young CM, He R, Emlet RB, Li Y, Qian H, Arellano SM, et al. Dispersal of deep-sea larvae from the intra-American seas: simulations of trajectories using ocean models. Integrative and Comparative Biology. 2012; 52(4): 483–496. doi: 10.1093/icb/ics090 22669174

[100] Cordeiro TA, Brandini FP, Rosa RS, and Sassi R. Deep Chlorophyll maximum in Western Equatorial Atlantic-How does it interact with islands slopes and seamounts. Marine Science. 2013; 3(1):30–37. doi: 10.5923/j.ms.20130301.03

[101] Luck C, Mejri S, Lewis J, Wills PS, Riche M, Shenker J, et al. Seasonal and spatial changes in sex hormone levels and oocyte development of bonefish (*Albula vulpes*). Environmental Biology of Fishes. 2019; 102(2):209–219. doi: 10.1007/s10641-018-0829-9

[102] Sherman K, Smith W, Morse W, Berman M, Green J, and Ejsymont L. Spawning strategies of fishes in relation to circulation, phytoplankton production, and pulses in zooplankton off the northeastern United States. Marine Ecology Progress Series. 1984; 18(1):1–9.

[103] Marshall DJ, Monro K, Bode M, Keough MJ, and Swearer, S. Phenotype–environment mismatches reduce connectivity in the sea. Ecology Letters. 2010; 13(1):128–140. doi: 10.1111/j.1461-0248.2009.01408.x 19968695

[104] Haak CR, Cowles GW, and Danylchuk AJ. Wave and tide-driven flow act on multiple scales to shape the distribution of a juvenile fish (*Albula vulpes*) in shallow nearshore habitats. Limnology and Oceanography. 2019; 64(2):597–615. doi: 10.1002/lno.11063

[105] McCormick MI. Delayed metamorphosis of a tropical reef fish (*Acanthurus triostegus*): a field experiment. Marine Ecology Progress Series. 1999; 176:25–38. doi: 10.3354/meps176025

[106] Smith KA, and Sinerchia M. Timing of recruitment events, residence periods and post-settlement growth of juvenile fish in a seagrass nursery area, south-eastern Australia. Environmental Biology of Fishes. 2004; 71(1):73–84. doi: 10.1023/B:EBFI.0000043154.96933.de

[107] Douglas MR, Chafin TK, Claussen JE, Philipp DP, and Douglas ME. Are populations of economically important bonefish and queen conch ’open’ or ’closed’ in the northern caribbean basin?. Marine Ecology. 2021; 42(2):e12639. doi: 10.1111/maec.12639

[108] Layman CA, and Silliman BR. Preliminary survey and diet analysis of juvenile fishes of an estuarine creek on Andros Island, Bahamas. Bulletin of Marine Science. 2002; 70(1):199–210.

[109] Pittman K, Yúfera M, Pavlidis M, Geffen AJ, Koven W, Ribeiro L, et al. Fantastically plastic: fish larvae equipped for a new world. Reviews in Aquaculture. 2013; 5:S224–S267. doi: 10.1111/raq.12034

[110] Marshall DJ, Monro K, Bode M, Keough MJ, and Swearer S. Phenotype-environment mismatches reduce connectivity in the sea. Ecology Letters. 2010; 13(1):128–140. doi: 10.1111/j.1461-0248.2009.01408.x 19968695

[111] Fronts Bakun A. and eddies as key structures in the habitat of marine fish larvae: opportunity, adaptive response and competitive advantage. Scientia Marina. 2006; 70(S2):105–22. doi: 10.3989/scimar.2006.70s2105

[112] McManus MA, and Woodson CB. Plankton distribution and ocean dispersal. Journal of Experimental Biology. 2012; 215(6):1008–1016. doi: 10.1242/jeb.059014 22357594

[113] Dufois F, Hardman-Mountford NJ, Greenwood J, Richardson AJ, Feng M, and Matear RJ. Anticyclonic eddies are more productive than cyclonic eddies in subtropical gyres because of winter mixing. Science Advances. 2016; 2(5):e1600282. doi: 10.1126/sciadv.1600282 27386549 PMC4928944

[114] Hoey AS, and McCormick MI. Selective predation for low body condition at the larval-juvenile transition of a coral reef fish. Oecologia. 2004; 139(1):23–29. doi: 10.1007/s00442-004-1489-3 14767752

[115] Shulman MJ, Ogden JC, Ebersole JP, McFarland WN, Miller SL, and Wolf NG. Priority effects in the recruitment of juvenile coral reef fishes. Ecology. 1983; 64(6):1508–1513. doi: 10.2307/1937505

[116] Almany GR. Priority effects in coral reef fish communities. Ecology. 2003; 84(7):1920–1935. doi: 10.1890/0012-9658(2003)084[1920:PEICRF]2.0.CO;2

[117] Poulos DE, and McCormick MI. Asymmetries in body condition and order of arrival influence competitive ability and survival in a coral reef fish. Oecologia. 2015; 179(3):719–728. doi: 10.1007/s00442-015-3401-8 26220881

[118] Haak CR, Hui FK, Cowles GW, and Danylchuk AJ. Positive interspecific associations consistent with social information use shape juvenile fish assemblages. Ecology. 2020; 101(2):e02920. doi: 10.1002/ecy.2920 31661156

[119] Szekeres P, Haak CR, Wilson AD, Danylchuk AJ, Brownscombe JW, Shultz AD, et al. Juvenile bonefish (*Albula vulpes*) show a preference to shoal with mojarra (*Eucinostomus* spp.) in the presence of conspecifics and another gregarious co-occurring species. Journal of Experimental Marine Biology and Ecology. 2020; 527:151374. doi: 10.1016/j.jembe.2020.151374

[120] Doostmohammadi A, Stocker R, and Ardekani AM. Low-Reynolds-number swimming at pycnoclines. Proceedings of the National Academy of Sciences. 2012; 109(10):3856–3861. doi: 10.1073/pnas.1116210109 22355147 PMC3309774

[121] Nickols KJ, Gaylord B, and Largier JL. The coastal boundary layer: predictable current structure decreases alongshore transport and alters scales of dispersal. Marine Ecology Progress Series. 2012; 464:17–35. doi: 10.3354/meps09875

[122] Corredor JE, and Morell JM. Seasonal variation of physical and biogeochemical features in eastern Caribbean Surface Water. Journal of Geophysical Research: Oceans. 2001; 106(C3):4517–4525. doi: 10.1029/2000JC000291

[123] Mejri S, Adams AJ, Shenker JM, Cianciotto AC, Robinson C, Uribe V, et al. Lipid composition and utilization in early stage leptocephalus larvae of Bonefish (*Albula vulpes*). Lipids. 2021; 56(1):81–91. doi: 10.1002/lipd.12278 32885865

[124] Uribe V, Wills PS, Shenker JM, Adams AJ, and Mejri S. A comprehensive biochemical characterization of settlement stage leptocephalus larvae of bonefish (*Albula vulpes*). Journal of Fish Biology. 2021; 99(6):1778–1785. doi: 10.1111/jfb.14846 34254307

[125] McDonnell AM, and Buesseler KO. Variability in the average sinking velocity of marine particles. Limnology and Oceanography. 2010; 55(5):2085–2096. doi: 10.4319/lo.2010.55.5.2085

[126] Sharqawy MH, Lienhard JH, and Zubair SM. Thermophysical properties of seawater: a review of existing correlations and data. Desalination and Water Treatment. 2010; 16(1–3):354–380. doi: 10.5004/dwt.2010.1079

[127] Okamura A, Yamada Y, Horie N, Utoh T, Mikawa N, Tanaka S, et al. Effects of water temperature on early development of Japanese eel *Anguilla japonica*. Fisheries Science. 2007; 73(6):1241–1248. doi: 10.1111/j.1444-2906.2007.01461.x

[128] McCormick MI, and Molony BW. Influence of water temperature during the larval stage on size, age and body condition of a tropical reef fish at settlement. Marine Ecology Progress Series. 1995; 118(1):59–68. doi: 10.3354/meps118059

[129] The Nature Conservancy. Caribbean Protected Areas 05052022_WFL1. 2022. [Online]. https://www.arcgis.com/apps/mapviewer/index.html?layers=6d15ba7e11a84278a13b40c8424db850. [Accessed July 2022].

