# Supplementary material for: Biophysical larval dispersal models of observed bonefish (Albula vulpes) spawning events in Abaco, The Bahamas: An assessment of population connectivity and ocean dynamics
Source: PLoS One. 2022 Oct 20;17(10):e0276528. doi: 10.1371/journal.pone.0276528 (PMC9584404; doi:10.1371/journal.pone.0276528)

**S1 Information**

The parameterization of *Albula vulpes* density over development through an equivalency to *Anguilla japonica* density over development.

No larval density measurements exist for bonefish. However, personal observations (Jonathan M. Shenker and Paul S. Wills) have noted sinking larvae as oil droplets decrease in size. This is to be expected due to the decrease in buoyant oil mass and total lipid content, driven by consumption of wax esters and steryl esters, leaving less buoyant triacylglycerols to comprise larval tissue [1, 2]. Density measurements over development have been taken for another Elopomorph, the Japanese eel *Anguilla japonica*, which we applied to bonefish [3]. The measured density of individual *A. japonica* were converted to a proportion in relation to the density of water at the upper thermocline. Furthermore, the length of each individual *A. japonica* can be converted into a proportion of the time duration of larval development. Therefore, each point in *Figure 5* of Tsukamoto et al. [3] was converted from *length*/*density* to *proportion of development time*/*larval density* *as a proportion of* *seawater density*). These proportions were transformed to fit the larval duration of bonefish.

The egg density of bonefish was parameterized to be of equal proportion to the thermocline density in Abaco as the average density of *A. japonica* was to the reported upper thermocline water density in Tsukamoto et al. [3]. The density of early-stage bonefish leptocephali during the oil droplet consumption phase showed a linear increase between the egg density and the average time of observed oil droplet consumption times: Halstead et al. [4] 56 hours post-hatch (hph) and Mejri et al. [2] 76 hph. Within the larval dispersal model, the oil droplet consumption time was estimated to be the average at 66 hph. The calculation for the rate-change is based on the proportion *average A. japonica larval density during oil droplet consumption*/*thermocline water density*. The *A. japonica* larval densities during the oil droplet consumption phase are inclusive of the continuum from hatching to complete consumption, as such, the *A. japonica* density proportion was a product of the average over the continuum and is representative of the middle time point of oil droplet consumption. The *A. japonica* density proportion is translationally associated with the halfway point of oil droplet consumption in *A. vulpes*, 46 hph, and the rate-change in bonefish density through the oil droplet consumption phase is calculated as the linear increase in density from the hatching density to the bonefish density that would be equally proportional to the thermocline density in Abaco on 12 Nov 2019 06:00 EST as the average *A. japonica* density is to the reported thermocline in Tsukamoto et al. [3]. The rate change was then applied from the hatching point through the entire oil droplet consumption phase, 25 hph to 66 hph.

The density of feeding bonefish leptocephali was again calculated as a linear increase, from the end of the oil droplet consumption phase up to the metamorphosing settlement stage leptocephalus. The linear increase is calculated by the linear equation derived from the *A. japonica* *proportion of larval duration* ~ *larval density*/*thermocline water density*:

$$Bonefish Density=\left( -0.0041* \frac{hph}{bonefish PLD}+1.0026 \right)*Thermocline Density$$

The equation returned an R^2^ = 0.6378. The density of settlement stage bonefish leptocephali was set in equal proportion to the observed thermocline water density (1.02409 g·cm^-3^) as the *A. japonica* larval density is in proportion to the upper thermocline density. The bonefish settlement stage begins at 41 days post-hatch and is maintained throughout the full 71 day PLD [5]. However, the larval transport model is parameterized to bring larvae up within the top 2 m of the surface from 41 days post-hatch onward (Vertical migration parameter enabled), as most settlement larvae were caught in the top 1 m of water [5]. Therefore, the lack of a more rigorous estimation of larval density during settlement does not impact the biological accuracy of the larval density parameter.

See below for bonefish larval density schedule.

**References**

1. Deibel D, Parrish CC, Grønkjær P, Munk P, and Gissel Nielsen T. Lipid class and fatty acid content of the leptocephalus larva of tropical eels. Lipids. 2012; 47:623–634. doi: 10.1007/s11745-012-3670-5.
2. Mejri S, Adams AJ, Shenker JM, Cianciotto AC, Robinson C, Uribe V, et al. Lipid composition and utilization in early stage leptocephalus larvae of bonefish (*Albula vulpes*). Lipids. 2021; 56(1):81–91. doi: 10.1002/lipd.12278.
3. Tsukamoto K, Yamada Y, Okamura A, Kaneko T, Tanaka H, Miller MJ, et al. Positive buoyancy in eel leptocephali: an adaptation for life in the ocean surface layer. Marine Biology. 2009; 156(5):835–846. doi: 10.1007/s00227-008-1123-8.
4. Halstead WR, Mejri S, Cianciotto AC, Wills PS, Van Leeuwen TE, Adams AJ, et al. Induced spawning and embryonic and early larval development of bonefish (*Albula vulpes*). Journal of Fish Biology. 2020; 96(3):825–830. doi: 10.1111/jfb.14250.
5. Mojica Jr. R, Shenker JM, Harnden CW, and Wagner DE. Recruitment of bonefish, *Albula vulpes*, around Lee Stocking Island, Bahamas. Fisheries Bulletin-NOAA. 1995; 93:666–674.

S1 Information. Parameterized particle density schedule (black line) from 0 to 41 days post spawn, after which larvae were parameterized to move into the top 2 m of the water column. The thermocline at the time of spawning was observed using a Castaway CTD. The density of the thermocline was measured by the CTD (gray hashed line).


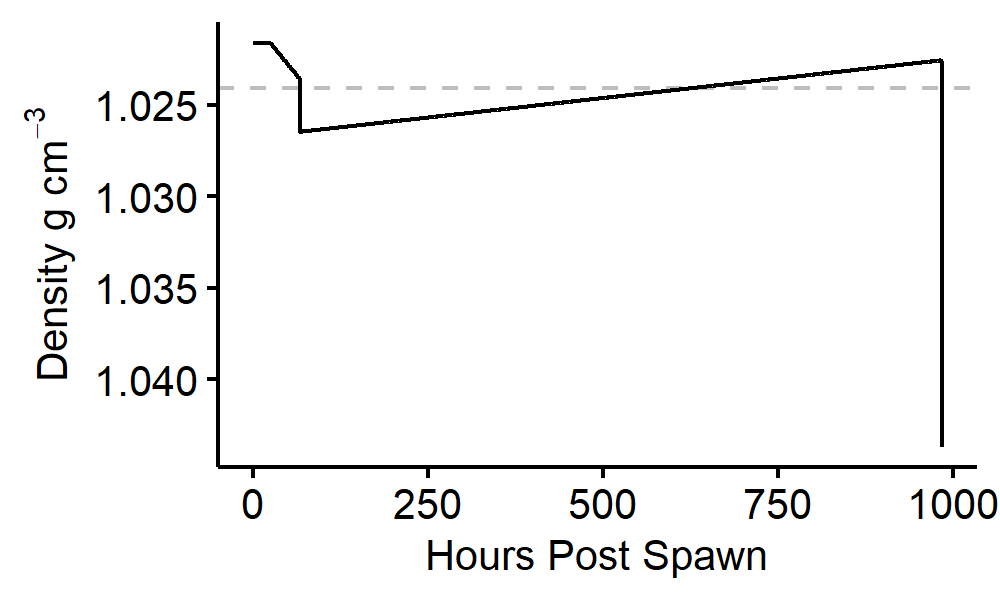

Supplement: S2 File — (DOCX) [file pone.0276528.s002.docx]
