# Supplementary material for: Biophysical larval dispersal models of observed bonefish (Albula vulpes) spawning events in Abaco, The Bahamas: An assessment of population connectivity and ocean dynamics
Source: PLoS One. 2022 Oct 20;17(10):e0276528. doi: 10.1371/journal.pone.0276528 (PMC9584404; doi:10.1371/journal.pone.0276528)

**S1 Figure**

S1 Fig A. Comparison of CTD data at the moment of spawning in 2019 (black line) with NCOM AmSeas temperature-depth data at the nearest time (-1 hr UTC) and location. NCOM AmSeas temperature-depth relationships at the next nearest longitude and latitude are included for spatial evaluation of measurement accuracy. CTD depths are limited to the 100 m depth rating of the Castaway CTD.


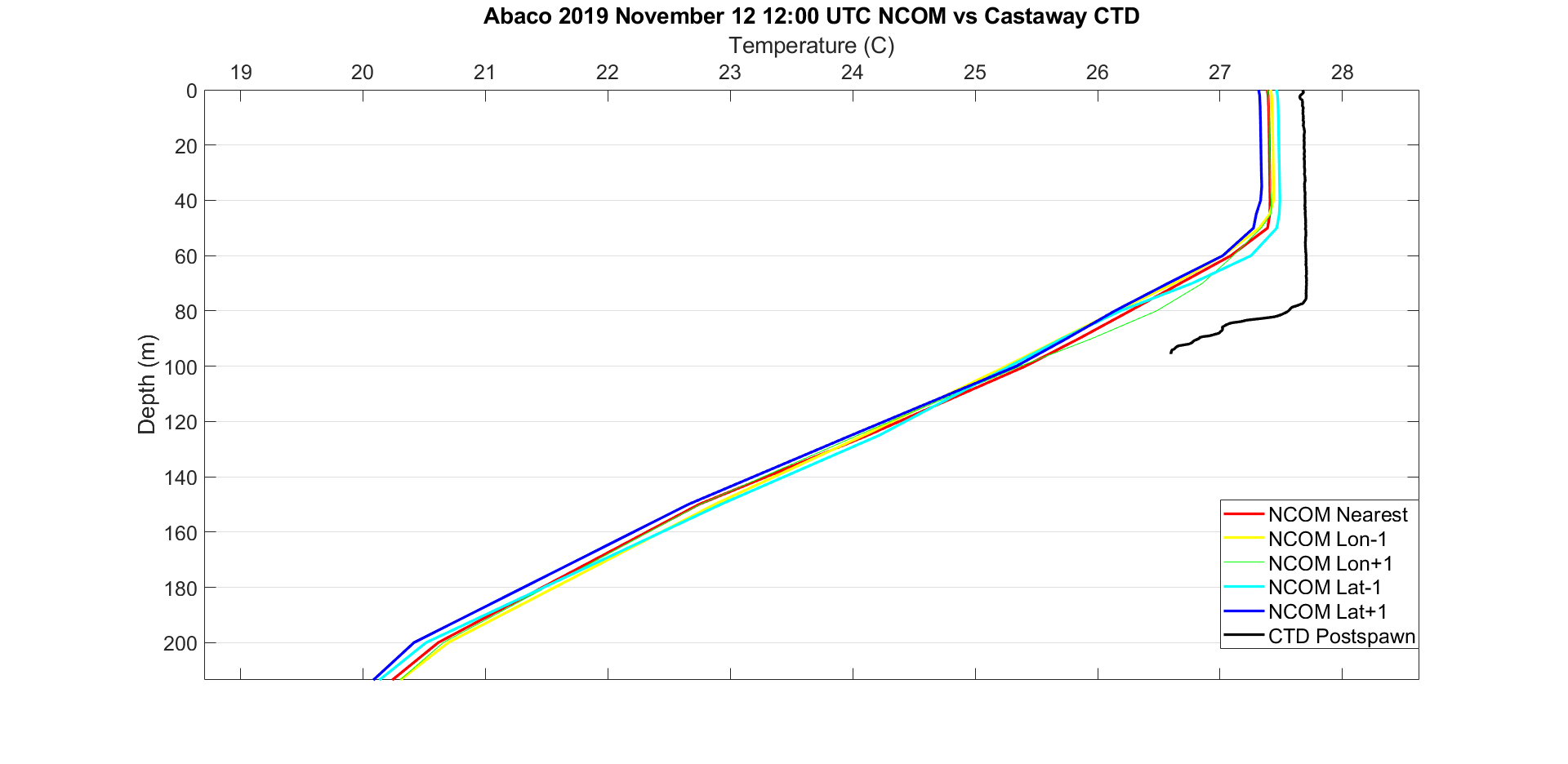


S1 Fig B. Root-Mean Squared Error (RMSE) of NCOM AmSeas and CTD temperature measurements. RMSE is calculated using NCOM AmSeas z depths paired to CTD depths with a minimized absolute difference in depth. Depths are limited to the 100 m depth rating of the Castaway CTD. 19 CTD casts taken were taken from 10 November 2019 to 12 November 2019. CTD casts were paired with the most spatiotemporally similar NCOM AmSeas data.


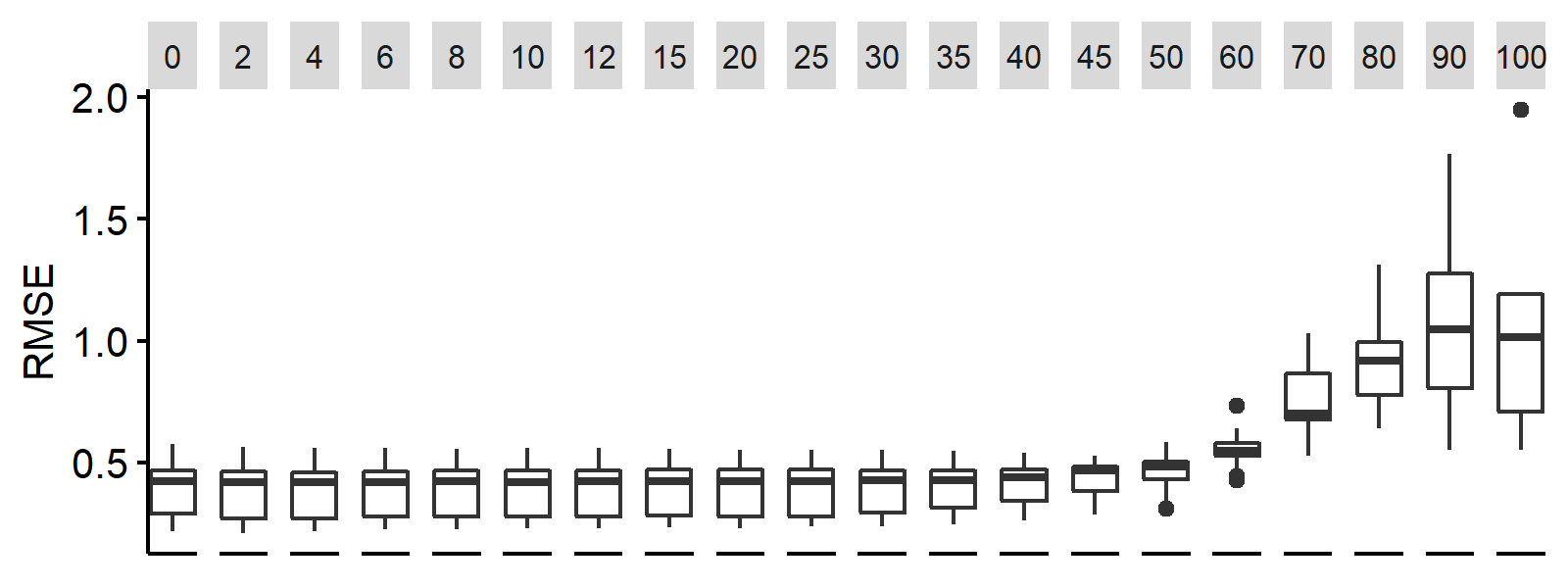

Supplement: S1 Fig — (DOCX) [file pone.0276528.s003.docx]
