# Supplementary material for: Biophysical larval dispersal models of observed bonefish (Albula vulpes) spawning events in Abaco, The Bahamas: An assessment of population connectivity and ocean dynamics
Source: PLoS One. 2022 Oct 20;17(10):e0276528. doi: 10.1371/journal.pone.0276528 (PMC9584404; doi:10.1371/journal.pone.0276528)
Supplement: S2 Fig — (DOCX) [file pone.0276528.s004.docx]

**S2 Figure**


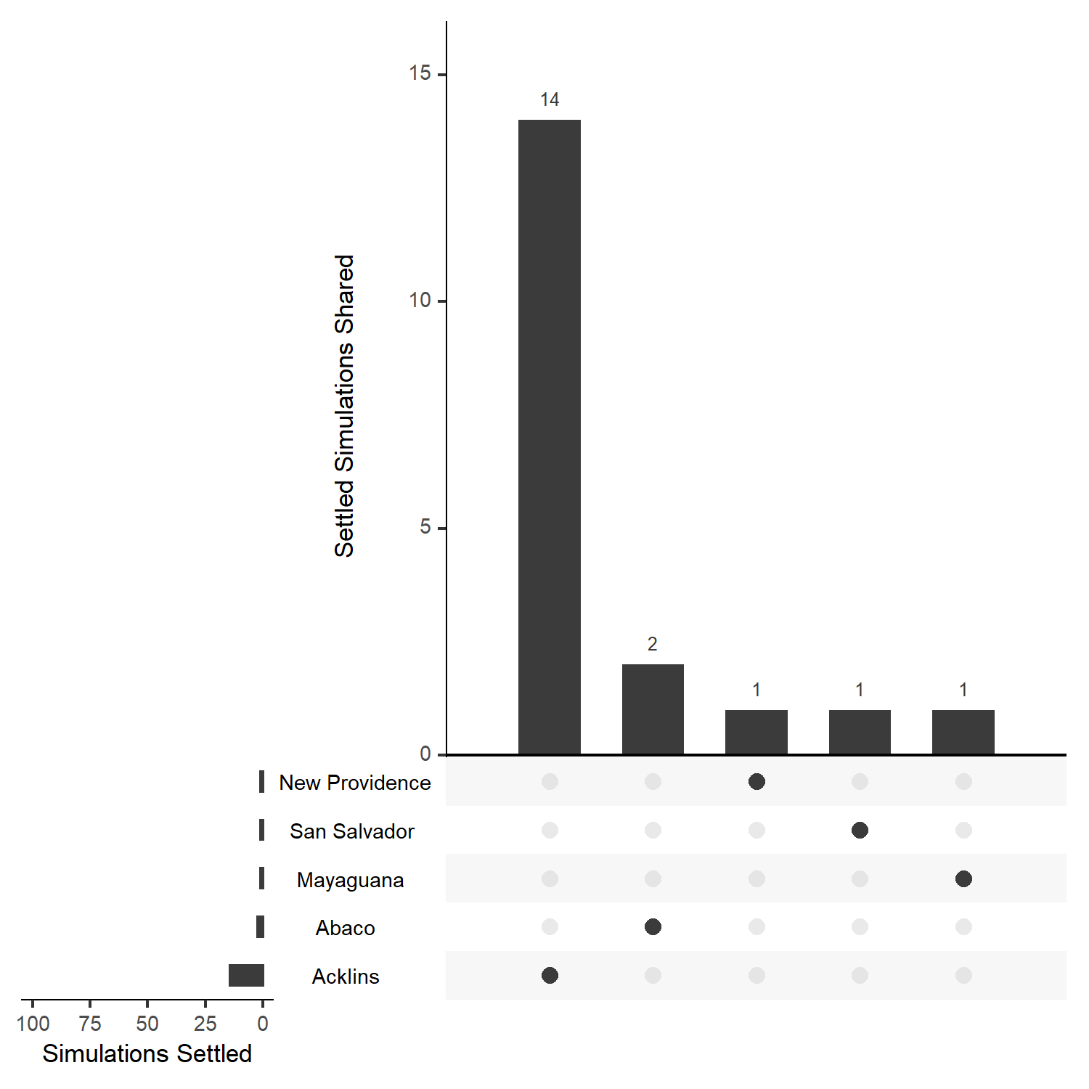


S2 Fig A. 2013 LDM iteration settlement footprints across 100 iterations. Footprints — combinations of islands settled — are indicated with black dots within the same column. The number of LDM iterations of a given settlement footprint is shown within the vertical bar plot. The number of LDM iterations in which an island was settled is shown within the horizontal bar plot..


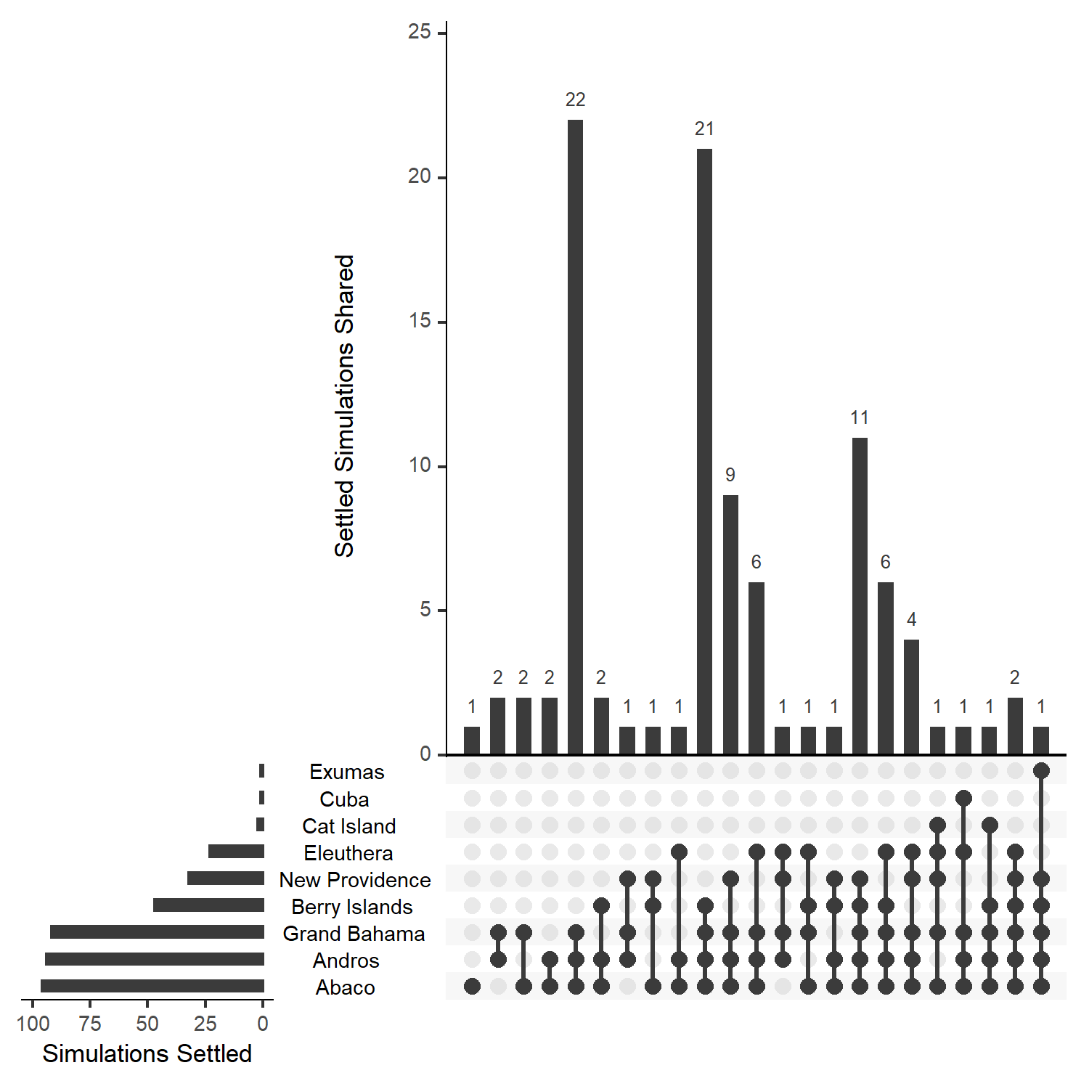


S2 Fig B. 2018 LDM iteration settlement footprints across 100 iterations. Footprints — combinations of islands settled — are indicated with black dots within the same column. The number of LDM iterations of a given settlement footprint is shown within the vertical bar plot. The number of LDM iterations in which an island was settled is shown within the horizontal bar plot..


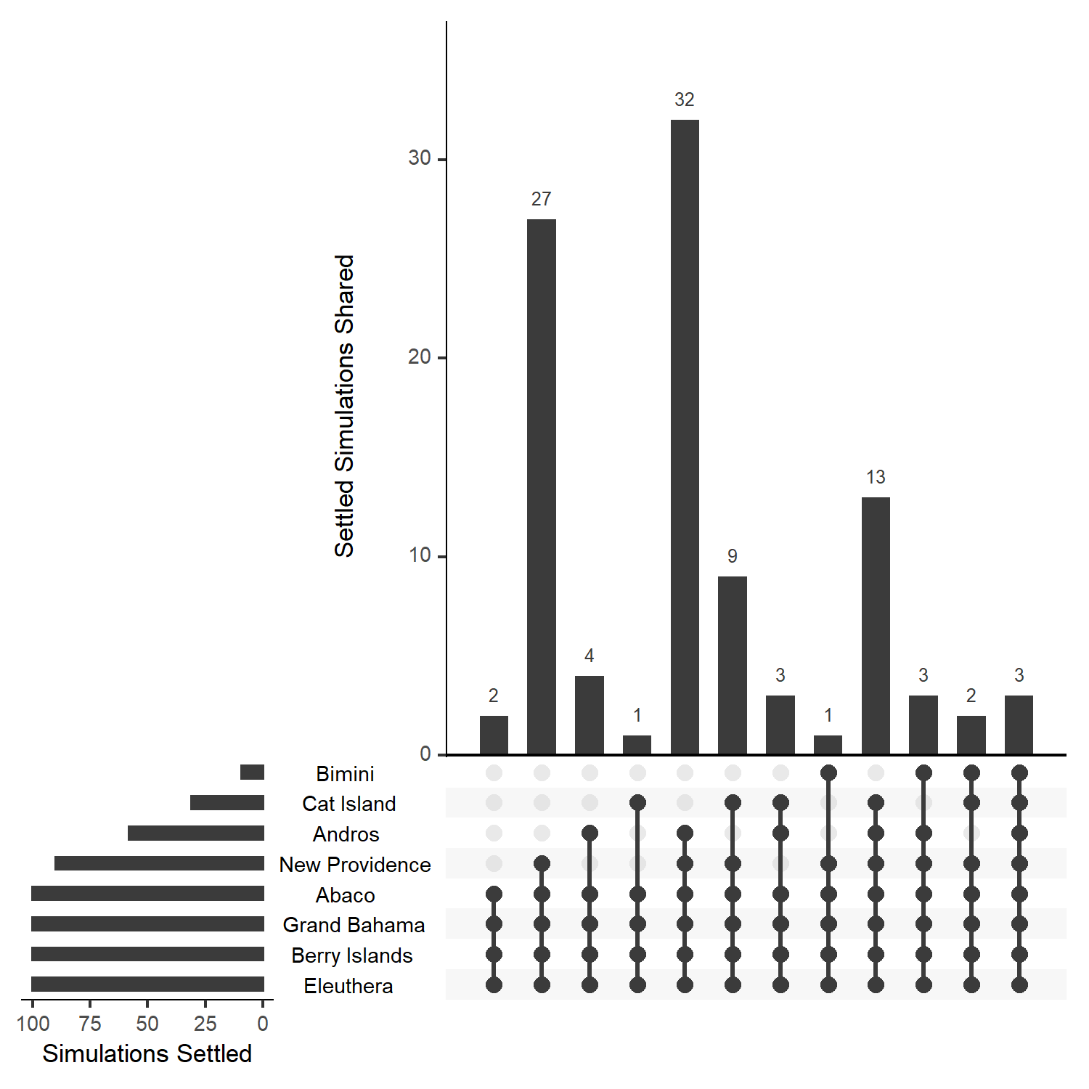


S2 Fig C. 2019 LDM iteration settlement footprints across 100 iterations. Footprints — combinations of islands settled — are indicated with black dots within the same column. The number of LDM iterations of a given settlement footprint is shown within the vertical bar plot. The number of LDM iterations in which an island was settled is shown within the horizontal bar plot..


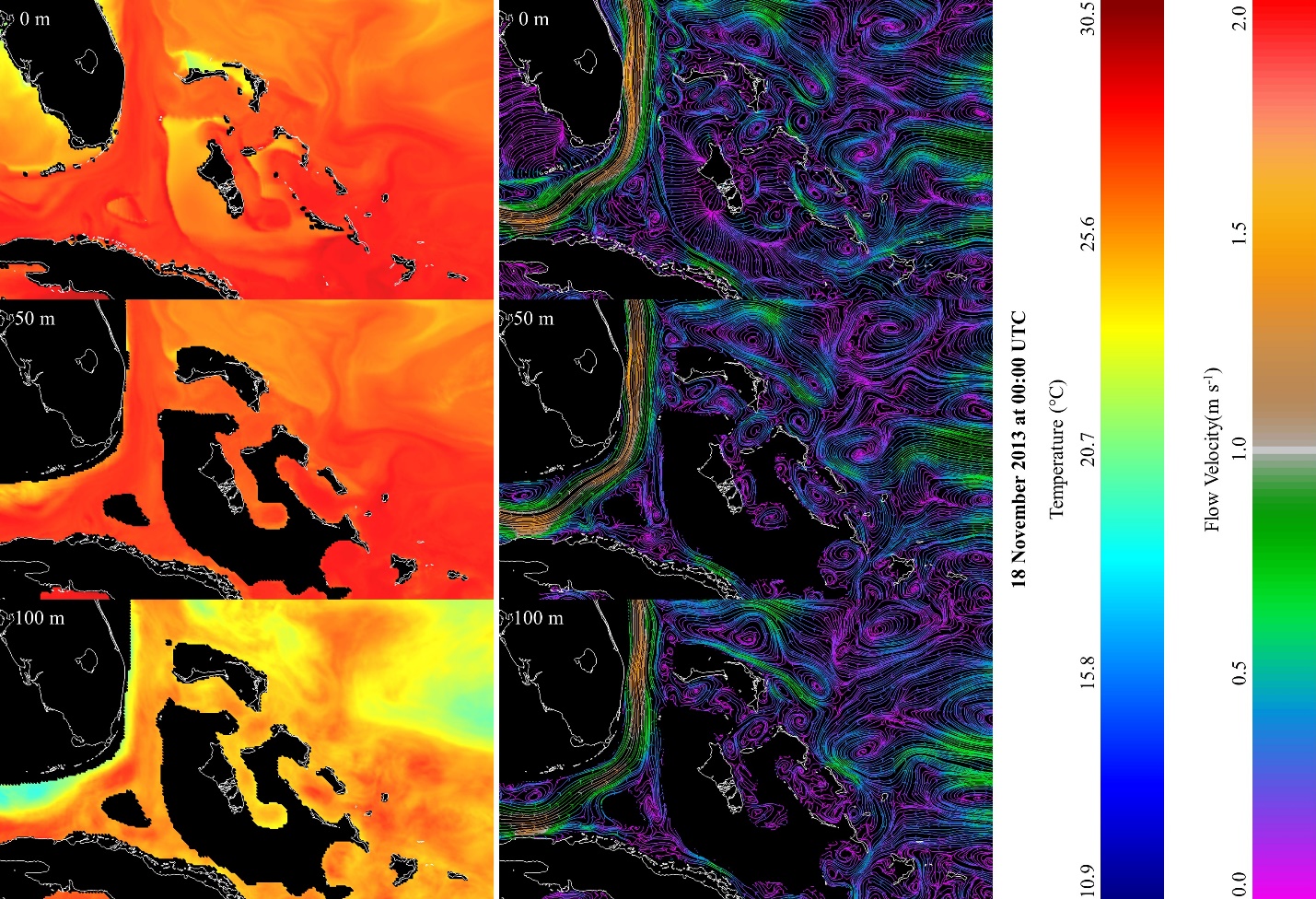


S2 Fig D. NCOM AmSeas thermal (left) and current (right) conditions at the time of spawning in 2013. Conditions shown are surface (0 m), the top extent of the spawning rush (50 m), and the bottom extent of the spawning rush (150 m).


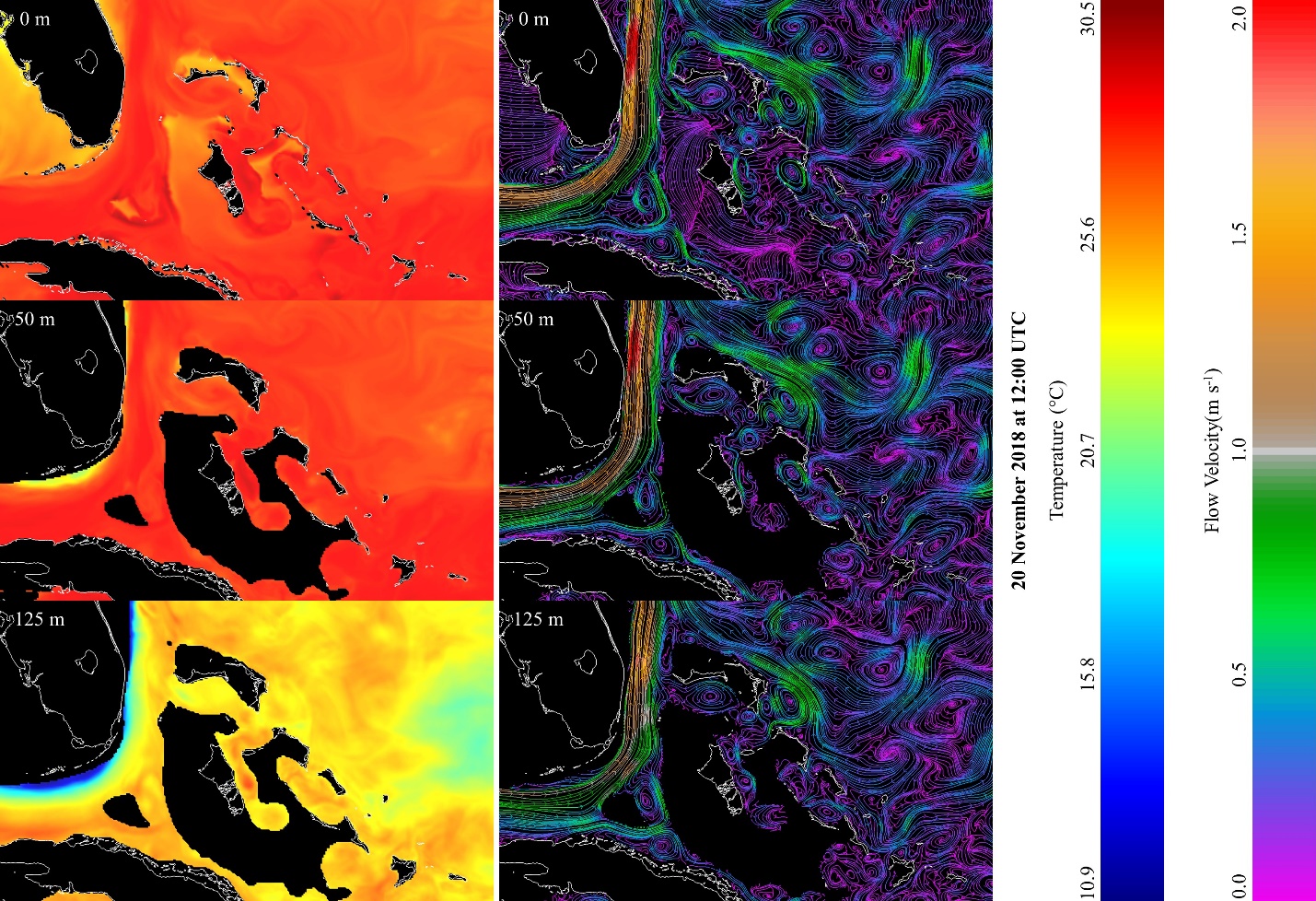


S2 Fig E. NCOM AmSeas thermal (left) and current (right) conditions at the time of spawning in 2018. Conditions shown are surface (0 m), the top extent of the spawning rush (50 m), and the bottom extent of the spawning rush (125 m).


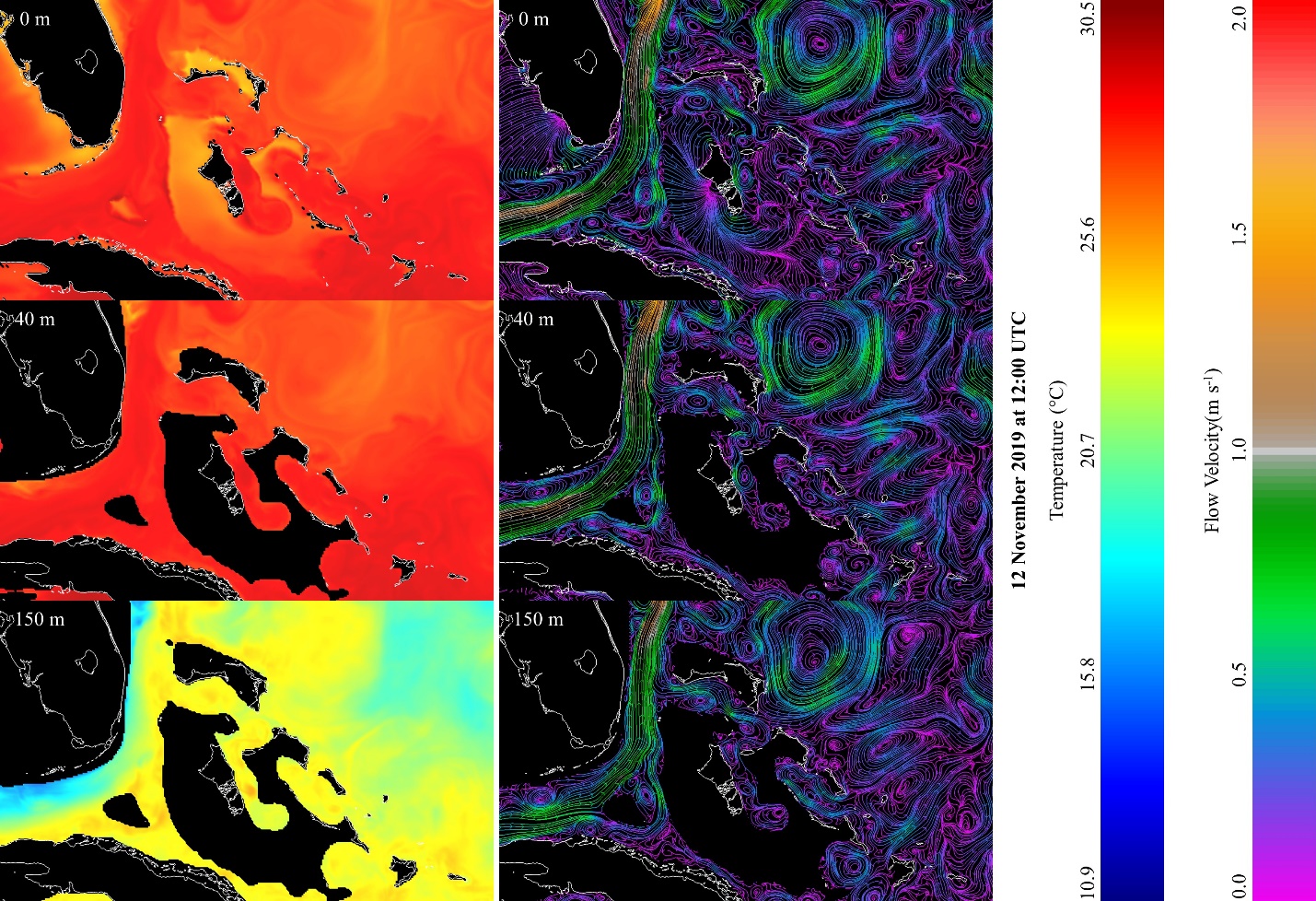


S2 Fig F. NCOM AmSeas thermal (left) and current (right) conditions at the time of spawning in 2019. Conditions shown are surface (0 m), the top extent of the spawning rush (40 m), and the bottom extent of the spawning rush (150 m).


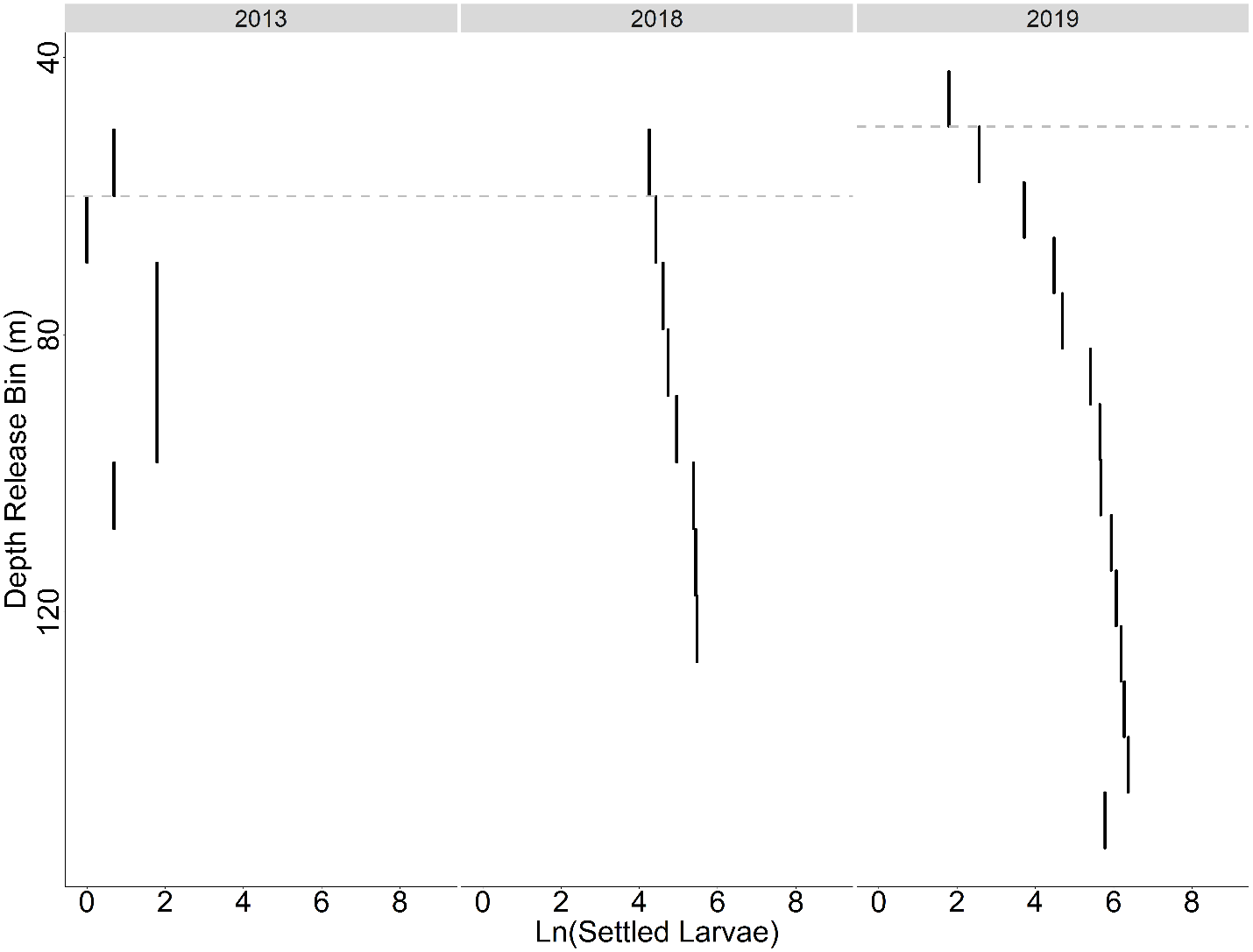


S2 Fig G. Settled larvae per 10 m binned release depth. The gray hashed line indicates the NCOM AmSeas thermocline at the location of the spawning events. Release depth bins were initialized at a depth 10 m shallower than the thermocline and are inclusive of the bottom depth.
