# Supplementary material for: Biophysical larval dispersal models of observed bonefish (Albula vulpes) spawning events in Abaco, The Bahamas: An assessment of population connectivity and ocean dynamics
Source: PLoS One. 2022 Oct 20;17(10):e0276528. doi: 10.1371/journal.pone.0276528 (PMC9584404; doi:10.1371/journal.pone.0276528)
Supplement: S1 Table — (DOCX) [file pone.0276528.s005.docx]

**S1 Table**

Results of Kruskall-Wallis Test and Dunn’s Test with Bonferroni Correction for comparing metrics among settled islands within years, and interannual relative relationships. Results are relative to Contrast heading (i.e., negative = less than heading island), with significant relationships bolded. ɑ = 0.05, with Bonferroni Correction significant when p ≤ ɑ/2.

1. 2018 settled island Coefficient of Dispersal Kruskal-Wallis Test and Dunn’s Test with Bonferroni Correction.

|  | Df | H | p-Val |
| --- | --- | --- | --- |
| **Island** | **8** | **286.793** | **0** |

| Contrast | Z | Adj p-Val |
| --- | --- | --- |
| *Abaco* |  |  |
| **Andros** | **-6.622** | **0.000** |
| **Berry Islands** | **6.039** | **0.000** |
| Cat Island | -2.878 | 0.072 |
| Cuba | -2.080 | 0.676 |
| **Eleuthera** | **3.426** | **0.011** |
| Exumas | -0.289 | 1.000 |
| **Grand Bahama** | **-9.850** | **0.000** |
| **New Providence** | **4.075** | **0.001** |
| *Andros* |  |  |
| **Berry Islands** | **10.015** | **0.000** |
| Cat Island | -2.132 | 0.594 |
| Cuba | -1.550 | 1.000 |
| **Eleuthera** | **6.079** | **0.000** |
| Exumas | 0.241 | 1.000 |
| **Grand Bahama** | **-3.238** | **0.022** |
| **New Providence** | **7.251** | **0.000** |
| *Berry Islands* |  |  |
| **Cat Island** | **-4.033** | **0.001** |
| Cuba | -2.915 | 0.064 |
| Eleuthera | -0.649 | 1.000 |
| Exumas | -1.136 | 1.000 |
| **Grand Bahama** | **-11.822** | **0.000** |
| New Providence | -0.724 | 1.000 |
| *Cat Island* |  |  |
| Cuba | -0.033 | 1.000 |
| Eleuthera | 3.742 | 0.003 |
| Exumas | 1.431 | 1.000 |
| Grand Bahama | 1.807 | 1.000 |
| **New Providence** | **3.788** | **0.003** |
| *Cuba* |  |  |
| Eleuthera | 2.735 | 0.113 |
| Exumas | 1.269 | 1.000 |
| Grand Bahama | 1.320 | 1.000 |
| New Providence | 2.753 | 0.106 |
| *Eleuthera* |  |  |
| Exumas | -0.974 | 1.000 |
| **Grand Bahama** | **-7.256** | **0.000** |
| New Providence | 0.009 | 1.000 |
| *Exumas* |  |  |
| Grand Bahama | -0.472 | 1.000 |
| New Providence | 0.982 | 1.000 |
| *Grand Bahama* |  |  |
| **New Providence** | **8.673** | **0.000** |

1. 2019 settled island Coefficient of Dispersal Kruskal-Wallis Test and Dunn’s Test with Bonferroni Correction.

|  | Df | H | p-Val |
| --- | --- | --- | --- |
| **Island** | **7** | **2030.3** | **<0.01** |

| Contrast | Z | Adj p-Val |
| --- | --- | --- |
| *Abaco* |  |  |
| **Andros** | **-13.506** | **0.000** |
| **Berry Islands** | **-5.345** | **0.000** |
| **Bimini** | **-8.058** | **0.000** |
| **Cat Island** | **-14.284** | **0.000** |
| **Eleuthera** | **-8.934** | **0.000** |
| **Grand Bahama** | **-40.678** | **0.000** |
| **New Providence** | **-14.739** | **0.000** |
| *Andros* |  |  |
| **Berry Islands** | **10.813** | **0.000** |
| Bimini | -2.779 | 0.076 |
| **Cat Island** | **-4.267** | **0.000** |
| **Eleuthera** | **8.852** | **0.000** |
| Grand Bahama | -2.374 | 0.246 |
| **New Providence** | **4.239** | **0.000** |
| *Berry Islands* |  |  |
| **Bimini** | **-7.130** | **0.000** |
| **Cat Island** | **-12.489** | **0.000** |
| **Eleuthera** | **-3.521** | **0.006** |
| **Grand Bahama** | **-29.299** | **0.000** |
| **New Providence** | **-9.969** | **0.000** |
| *Bimini* |  |  |
| Cat Island | 0.129 | 1.000 |
| **Eleuthera** | **6.404** | **0.000** |
| Grand Bahama | 2.038 | 0.582 |
| **New Providence** | **4.647** | **0.000** |
| *Cat Island* |  |  |
| **Eleuthera** | **11.139** | **0.000** |
| **Grand Bahama** | **3.406** | **0.009** |
| **New Providence** | **7.860** | **0.000** |
| *Eleuthera* |  |  |
| **Grand Bahama** | **-23.573** | **0.000** |
| **New Providence** | **-6.805** | **0.000** |
| *Grand Bahama* |  |  |
| **New Providence** | **11.566** | **0.000** |

1. 2018 settled island days post spawn (DPS) Kruskal-Wallis Test and Dunn’s Test with Bonferroni Correction.

|  | Df | H | p-Val |
| --- | --- | --- | --- |
| **Island** | **8** | **37.129** | **<0.01** |

| Contrast | Z | Adj p-Val |
| --- | --- | --- |
| *Abaco* |  |  |
| Andros | -0.184 | 1.000 |
| Berry Islands | 2.533 | 0.204 |
| Cat Island | 2.047 | 0.733 |
| Cuba | -1.511 | 1.000 |
| Eleuthera | -0.903 | 1.000 |
| Exumas | -1.046 | 1.000 |
| Grand Bahama | 3.018 | 0.046 |
| New Providence | -2.164 | 0.548 |
| *Andros* |  |  |
| Berry Islands | 2.698 | 0.126 |
| Cat Island | 2.069 | 0.693 |
| Cuba | -1.497 | 1.000 |
| Eleuthera | -0.840 | 1.000 |
| Exumas | -1.032 | 1.000 |
| **Grand Bahama** | **3.479** | **0.009** |
| New Providence | -2.110 | 0.627 |
| *Berry Islands* |  |  |
| Cat Island | 1.522 | 1.000 |
| Cuba | -1.858 | 1.000 |
| Eleuthera | -2.323 | 0.364 |
| Exumas | -1.396 | 1.000 |
| Grand Bahama | -0.914 | 1.000 |
| **New Providence** | **-3.549** | **0.007** |
| *Cat Island* |  |  |
| Cuba | -2.422 | 0.278 |
| Eleuthera | -2.232 | 0.461 |
| Exumas | -2.042 | 0.741 |
| Grand Bahama | -1.720 | 1.000 |
| New Providence | -2.519 | 0.212 |
| *Cuba* |  |  |
| Eleuthera | 1.304 | 1.000 |
| Exumas | 0.329 | 1.000 |
| Grand Bahama | 1.746 | 1.000 |
| New Providence | 1.125 | 1.000 |
| *Eleuthera* |  |  |
| Exumas | -0.847 | 1.000 |
| Grand Bahama | 2.074 | 0.685 |
| New Providence | -0.744 | 1.000 |
| *Exumas* |  |  |
| Grand Bahama | 1.280 | 1.000 |
| New Providence | 0.665 | 1.000 |
| *Grand Bahama* |  |  |
| **New Providence** | **-3.594** | **0.006** |

1. 2019 settled island days post spawn (DPS) Kruskal-Wallis Test and Dunn’s Test with Bonferroni Correction.

|  | Df | H | p-Val |
| --- | --- | --- | --- |
| **Island** | **7** | **346.71** | **<0.01** |

| Contrast | Z | Adj p-Val |
| --- | --- | --- |
| *Abaco* |  |  |
| **Andros** | **-4.648** | **0.000** |
| **Berry Islands** | **7.945** | **0.000** |
| **Bimini** | **4.151** | **0.001** |
| **Cat Island** | **-4.967** | **0.000** |
| **Eleuthera** | **-8.03** | **0.000** |
| Grand Bahama | -0.620 | 1.000 |
| **New Providence** | **-8.424** | **0.000** |
| *Andros* |  |  |
| **Berry Islands** | **7.936** | **0.000** |
| **Bimini** | **5.576** | **0.000** |
| Cat Island | -1.512 | 1.000 |
| Eleuthera | 0.432 | 1.000 |
| **Grand Bahama** | **4.370** | **0.000** |
| New Providence | -0.355 | 1.000 |
| *Berry Islands* |  |  |
| Bimini | 2.805 | 0.070 |
| **Cat Island** | **-7.273** | **0.000** |
| **Eleuthera** | **-14.393** | **0.000** |
| **Grand Bahama** | **-8.156** | **0.000** |
| **New Providence** | **-13.238** | **0.000** |
| *Bimini* |  |  |
| **Cat Island** | **-6.084** | **0.000** |
| **Eleuthera** | **-5.703** | **0.000** |
| **Grand Bahama** | **-4.24** | **0.000** |
| **New Providence** | **-5.965** | **0.000** |
| *Cat Island* |  |  |
| Eleuthera | 2.056 | 0.557 |
| **Grand Bahama** | **4.783** | **0.000** |
| New Providence | 1.449 | 1.000 |
| *Eleuthera* |  |  |
| **Grand Bahama** | **8.009** | **0.000** |
| New Providence | -1.259 | 1.000 |
| *Grand Bahama* |  |  |
| **New Providence** | **-7.840** | **0.000** |

1. 2018 settled island release depth Kruskal-Wallis Test and Dunn’s Test with Bonferroni Correction.

|  | Df | H | p-Val |
| --- | --- | --- | --- |
| Island | 8 | 13.79 | 0.087 |

| Contrast | Z | Adj p-Val |
| --- | --- | --- |
| *Abaco* |  |  |
| Andros | 1.648 | 1.000 |
| Berry Islands | 1.758 | 1.000 |
| Cat Island | 0.730 | 1.000 |
| Cuba | -0.156 | 1.000 |
| Eleuthera | 2.440 | 0.265 |
| Exumas | 0.950 | 1.000 |
| Grand Bahama | 0.088 | 1.000 |
| New Providence | -0.695 | 1.000 |
| *Andros* |  |  |
| Berry Islands | 0.843 | 1.000 |
| Cat Island | 0.544 | 1.000 |
| Cuba | -0.289 | 1.000 |
| Eleuthera | 1.815 | 1.000 |
| Exumas | 0.819 | 1.000 |
| Grand Bahama | -1.757 | 1.000 |
| New Providence | -1.480 | 1.000 |
| *Berry Islands* |  |  |
| Cat Island | 0.374 | 1.000 |
| Cuba | -0.402 | 1.000 |
| Eleuthera | 1.075 | 1.000 |
| Exumas | 0.697 | 1.000 |
| Grand Bahama | -1.770 | 1.000 |
| New Providence | -1.787 | 1.000 |
| *Cat Island* |  |  |
| Cuba | -0.551 | 1.000 |
| Eleuthera | -0.024 | 1.000 |
| Exumas | 0.354 | 1.000 |
| Grand Bahama | -0.721 | 1.000 |
| New Providence | -0.880 | 1.000 |
| *Cuba* |  |  |
| Eleuthera | 0.645 | 1.000 |
| Exumas | 0.784 | 1.000 |
| Grand Bahama | 0.163 | 1.000 |
| New Providence | 0.036 | 1.000 |
| *Eleuthera* |  |  |
| Exumas | 0.443 | 1.000 |
| Grand Bahama | -2.446 | 0.260 |
| New Providence | -2.440 | 0.264 |
| *Exumas* |  |  |
| Grand Bahama | -0.944 | 1.000 |
| New Providence | -1.058 | 1.000 |
| *Grand Bahama* |  |  |
| New Providence | -0.751 | 1.000 |

1. 2019 settled island release depth Kruskal-Wallis Test and Dunn’s Test with Bonferroni Correction.

|  | Df | H | p-Val |
| --- | --- | --- | --- |
| Island | 7 | 10.397 | 0.167 |

| Contrast | Z | Adj p-Val |
| --- | --- | --- |
| *Abaco* |  |  |
| Andros | 1.377 | 1.000 |
| Berry Islands | 0.829 | 1.000 |
| Bimini | 1.855 | 0.891 |
| Cat Island | -0.958 | 1.000 |
| Eleuthera | -0.681 | 1.000 |
| Grand Bahama | -0.864 | 1.000 |
| New Providence | 0.844 | 1.000 |
| *Andros* |  |  |
| Berry Islands | -0.980 | 1.000 |
| Bimini | 1.258 | 1.000 |
| Cat Island | -1.602 | 1.000 |
| Eleuthera | -1.629 | 1.000 |
| Grand Bahama | -1.700 | 1.000 |
| New Providence | -0.788 | 1.000 |
| *Berry Islands* |  |  |
| Bimini | 1.708 | 1.000 |
| Cat Island | -1.192 | 1.000 |
| Eleuthera | -1.291 | 1.000 |
| Grand Bahama | -1.528 | 1.000 |
| New Providence | 0.212 | 1.000 |
| *Bimini* |  |  |
| Cat Island | -2.102 | 0.498 |
| Eleuthera | -1.1965 | 0.692 |
| Grand Bahama | -1.980 | 0.668 |
| New Providence | -1.638 | 1.000 |
| *Cat Island* |  |  |
| Eleuthera | 0.721 | 1.000 |
| Grand Bahama | 0.724 | 1.000 |
| New Providence | 1.240 | 1.000 |
| *Eleuthera* |  |  |
| Grand Bahama | -0.0266 | 1.000 |
| New Providence | 1.238 | 1.000 |
| *Grand Bahama* |  |  |
| New Providence | 1.377 | 1.000 |

1. 2018 settled island mean dispersal depth Kruskal-Wallis Test and Dunn’s Test with Bonferroni Correction.

|  | Df | H | p-Val |
| --- | --- | --- | --- |
| **Island** | **8** | **529.12** | **<0.01** |

| Contrast | Z | Adj p-Val |
| --- | --- | --- |
| *Abaco* |  |  |
| **Andros** | **18.597** | **0.000** |
| **Berry Islands** | **3.237** | **0.022** |
| Cat Island | 0.076 | 1.000 |
| Cuba | 2.137 | 0.587 |
| Eleuthera | 0.502 | 1.000 |
| Exumas | -0.129 | 1.000 |
| Grand Bahama | 0.510 | 1.000 |
| New Providence | 0.401 | 1.000 |
| *Andros* |  |  |
| **Berry Islands** | **-7.463** | **0.000** |
| Cat Island | -2.027 | 0.767 |
| Cuba | 0.648 | 1.000 |
| **Eleuthera** | **-6.839** | **0.000** |
| Exumas | -1.618 | 1.000 |
| **Grand Bahama** | **-20.343** | **0.000** |
| **New Providence** | **-8.339** | **0.000** |
| *Berry Islands* |  |  |
| Cat Island | -0.563 | 1.000 |
| Cuba | 1.670 | 1.000 |
| Eleuthera | -1.518 | 1.000 |
| Exumas | -0.582 | 1.000 |
| Grand Bahama | -3.062 | 0.040 |
| New Providence | -1.882 | 1.000 |
| *Cat Island* |  |  |
| Cuba | 1.704 | 1.000 |
| Eleuthera | 0.067 | 1.000 |
| Exumas | -0.149 | 1.000 |
| Grand Bahama | -0.021 | 1.000 |
| New Providence | 0.021 | 1.000 |
| *Cuba* |  |  |
| Eleuthera | -2.000 | 0.819 |
| Exumas | -1.605 | 1.000 |
| Grand Bahama | -2.099 | 0.645 |
| New Providence | -2.045 | 0.736 |
| *Eleuthera* |  |  |
| Exumas | -0.227 | 1.000 |
| Grand Bahama | -0.315 | 1.000 |
| New Providence | -0.131 | 1.000 |
| *Exumas* |  |  |
| Grand Bahama | 0.168 | 1.000 |
| New Providence | 0.196 | 1.000 |
| *Grand Bahama* |  |  |
| New Providence | 0.178 | 1.000 |

1. 2019 settled island mean dispersal depth Kruskal-Wallis Test and Dunn’s Test with Bonferroni Correction.

|  | Df | H | p-Val |
| --- | --- | --- | --- |
| **Island** | **7** | **50.176** | **<0.01** |

| Contrast | Z | Adj p-Val |
| --- | --- | --- |
| *Abaco* |  |  |
| **Andros** | **3.162** | **0.022** |
| **Berry Islands** | **5.260** | **0.000** |
| Bimini | 1.331 | 1.000 |
| Cat Island | 1.661 | 1.000 |
| **Eleuthera** | **4.666** | **0.000** |
| **Grand Bahama** | **4.602** | **0.000** |
| **New Providence** | **4.186** | **0.000** |
| *Andros* |  |  |
| Berry Islands | -0.805 | 1.000 |
| Bimini | 0.126 | 1.000 |
| Cat Island | -0.422 | 1.000 |
| Eleuthera | -0.898 | 1.000 |
| Grand Bahama | -1.352 | 1.000 |
| New Providence | -0.594 | 1.000 |
| *Berry Islands* |  |  |
| Bimini | 0.446 | 1.000 |
| Cat Island | 0.066 | 1.000 |
| Eleuthera | -0.202 | 1.000 |
| Grand Bahama | -1.157 | 1.000 |
| New Providence | 0.266 | 1.000 |
| *Bimini* |  |  |
| Cat Island | -0.362 | 1.000 |
| Eleuthera | -0.485 | 1.000 |
| Grand Bahama | -0.649 | 1.000 |
| New Providence | -0.376 | 1.000 |
| *Cat Island* |  |  |
| Eleuthera | -0.138 | 1.000 |
| Grand Bahama | -0.430 | 1.000 |
| New Providence | 0.048 | 1.000 |
| *Eleuthera* |  |  |
| Grand Bahama | -0.862 | 1.000 |
| New Providence | 0.419 | 1.000 |
| *Grand Bahama* |  |  |
| New Providence | 1.154 | 1.000 |

1. 2018 settled island mean temperature Kruskal-Wallis Test and Dunn’s Test with Bonferroni Correction.

|  | Df | H | p-Val |
| --- | --- | --- | --- |
| **Island** | **8** | **534.88** | **<0.01** |

| Contrast | Z | Adj p-Val |
| --- | --- | --- |
| *Abaco* |  |  |
| **Andros** | **-21.317** | **0.000** |
| **Berry Islands** | **-10.031** | **0.000** |
| Cat Island | -2.484 | 0.234 |
| Cuba | -1.350 | 1.000 |
| Eleuthera | -2.441 | 0.264 |
| Exumas | -1.582 | 1.000 |
| **Grand Bahama** | **-6.743** | **0.000** |
| **New Providence** | **-4.834** | **0.000** |
| *Andros* |  |  |
| Berry Islands | 2.089 | 0.661 |
| Cat Island | -0.075 | 1.000 |
| Cuba | 0.357 | 1.000 |
| **Eleuthera** | **5.953** | **0.000** |
| Exumas | 0.125 | 1.000 |
| **Grand Bahama** | **16.641** | **0.000** |
| **New Providence** | **5.117** | **0.000** |
| *Berry Islands* |  |  |
| Cat Island | -0.476 | 1.000 |
| Cuba | 0.068 | 1.000 |
| **Eleuthera** | **3.926** | **0.002** |
| Exumas | -0.163 | 1.000 |
| **Grand Bahama** | **6.569** | **0.000** |
| New Providence | 2.824 | 0.085 |
| *Cat Island* |  |  |
| Cuba | 0.335 | 1.000 |
| Eleuthera | 1.720 | 1.000 |
| Exumas | 0.146 | 1.000 |
| Grand Bahama | 1.751 | 1.000 |
| New Providence | 1.276 | 1.000 |
| *Cuba* |  |  |
| Eleuthera | 0.835 | 1.000 |
| Exumas | -0.164 | 1.000 |
| Grand Bahama | 0.829 | 1.000 |
| New Providence | 0.509 | 1.000 |
| *Eleuthera* |  |  |
| Exumas | -1.063 | 1.000 |
| Grand Bahama | -0.103 | 1.000 |
| New Providence | -1.320 | 1.000 |
| *Exumas* |  |  |
| Grand Bahama | 1.061 | 1.000 |
| New Providence | 0.738 | 1.000 |
| *Grand Bahama* |  |  |
| New Providence | -1.862 | 1.000 |

1. 2019 settled island mean temperature Kruskal-Wallis Test and Dunn’s Test with Bonferroni Correction.

|  | Df | H | p-Val |
| --- | --- | --- | --- |
| **Island** | **7** | **524.29** | **<0.01** |

| Contrast | Z | Adj p-Val |
| --- | --- | --- |
| *Abaco* |  |  |
| **Andros** | **-8.482** | **0.000** |
| **Berry Islands** | **-19.410** | **0.000** |
| Bimini | -3.028 | 0.035 |
| **Cat Island** | **-5.237** | **0.000** |
| **Eleuthera** | **-11.111** | **0.000** |
| Grand Bahama | -2.588 | 0.135 |
| **New Providence** | **-9.309** | **0.000** |
| *Andros* |  |  |
| Berry Islands | -0.124 | 1.000 |
| Bimini | 0.172 | 1.000 |
| Cat Island | 0.472 | 1.000 |
| Eleuthera | 3.048 | 0.032 |
| **Grand Bahama** | **7.409** | **0.000** |
| New Providence | 2.634 | 0.118 |
| *Berry Islands* |  |  |
| Bimini | 0.230 | 1.000 |
| Cat Island | 0.637 | 1.000 |
| **Eleuthera** | **6.173** | **0.000** |
| **Grand Bahama** | **16.454** | **0.000** |
| **New Providence** | **4.697** | **0.000** |
| *Bimini* |  |  |
| Cat Island | 0.112 | 1.000 |
| Eleuthera | 1.016 | 1.000 |
| Grand Bahama | 2.642 | 0.112 |
| New Providence | 0.905 | 1.000 |
| *Cat Island* |  |  |
| Eleuthera | 1.584 | 1.000 |
| **Grand Bahama** | **4.529** | **0.000** |
| New Providence | 1.362 | 1.000 |
| *Eleuthera* |  |  |
| **Grand Bahama** | **8.680** | **0.000** |
| New Providence | -0.407 | 1.000 |
| *Grand Bahama* |  |  |
| **New Providence** | **-7.449** | **0.000** |
